# Electronic Health Diary Campaigns to Complement Longitudinal Assessments in Persons With Multiple Sclerosis: Nested Observational Study

**DOI:** 10.2196/38709

**Published:** 2022-10-05

**Authors:** Chloé Sieber, Deborah Chiavi, Christina Haag, Marco Kaufmann, Andrea B Horn, Holger Dressel, Chiara Zecca, Pasquale Calabrese, Caroline Pot, Christian Philipp Kamm, Viktor von Wyl, Swiss Multiple Sclerosis Registry

**Affiliations:** 1 Swiss Multiple Sclerosis Registry Epidemiology, Biostatistics and Prevention Institute University of Zurich Zurich Switzerland; 2 Institute for Implementation Science in Health Care University of Zurich Zürich Switzerland; 3 University Research Priority Program “Dynamics of Healthy Aging” University of Zurich Zurich Switzerland; 4 Competence Center of Gerontology University of Zurich Zurich Switzerland; 5 Department of Psychology University of Zurich Zurich Switzerland; 6 Division of Occupational and Environmental Medicine University of Zurich and University Hospital Zurich Zurich Switzerland; 7 Multiple Sclerosis Center Neurocenter of Southern Switzerland Ente Ospedaliero Cantonale Lugano Switzerland; 8 Faculty of Biomedical Sciences Università della Svizzera Italiana Lugano Switzerland; 9 Neuropsychology and Behavioral Neurology Unit Division of Molecular and Cognitive Neuroscience University of Basel Basel Switzerland; 10 Department of Neurology University Clinic of Basel Basel Switzerland; 11 Service of Neurology Department of Clinical Neurosciences Lausanne University Hospital and University of Lausanne Lausanne Switzerland; 12 Department of Neurology Inselspital Bern University Hospital and University of Bern Bern Switzerland; 13 Neurocenter Cantonal Hospital Lucerne Lucerne Switzerland; 14 See Acknowledgments

**Keywords:** registry, multiple sclerosis, digital health, electronic health diary, diary, participation, adherence, patient-reported outcome, natural language processing, unstructured text

## Abstract

**Background:**

Electronic health diaries hold promise in complementing standardized surveys in prospective health studies but are fraught with numerous methodological challenges.

**Objective:**

The study aimed to investigate participant characteristics and other factors associated with response to an electronic health diary campaign in persons with multiple sclerosis, identify recurrent topics in free-text diary entries, and assess the added value of structured diary entries with regard to current symptoms and medication intake when compared with survey-collected information.

**Methods:**

Data were collected by the Swiss Multiple Sclerosis Registry during a nested electronic health diary campaign and during a regular semiannual Swiss Multiple Sclerosis Registry follow-up survey serving as comparator. The characteristics of campaign participants were descriptively compared with those of nonparticipants. Diary content was analyzed using the Linguistic Inquiry and Word Count 2015 software (Pennebaker Conglomerates, Inc) and descriptive keyword analyses. The similarities between structured diary data and follow-up survey data on health-related quality of life, symptoms, and medication intake were examined using the Jaccard index.

**Results:**

Campaign participants (n=134; diary entries: n=815) were more often women, were not working full time, did not have a higher education degree, had a more advanced gait impairment, and were on average 5 years older (median age 52.5, IQR 43.25-59.75 years) than eligible nonparticipants (median age 47, IQR 38-55 years; n=524). Diary free-text entries (n=632; participants: n=100) most often contained references to the following standard Linguistic Inquiry and Word Count word categories: negative emotion (193/632, 30.5%), body parts or body functioning (191/632, 30.2%), health (94/632, 14.9%), or work (67/632, 10.6%). Analogously, the most frequently mentioned keywords (diary entries: n=526; participants: n=93) were “good,” “day,” and “work.” Similarities between diary data and follow-up survey data, collected 14 months apart (median), were high for health-related quality of life and stable for slow-changing symptoms such as fatigue or gait disorder. Similarities were also comparatively high for drugs requiring a regular application, including interferon beta-1a (Avonex) and glatiramer acetate (Copaxone), and for modern oral therapies such as fingolimod (Gilenya) and teriflunomide (Aubagio).

**Conclusions:**

Diary campaign participation seemed dependent on time availability and symptom burden and was enhanced by reminder emails. Electronic health diaries are a meaningful complement to regular structured surveys and can provide more detailed information regarding medication use and symptoms. However, they should ideally be embedded into promotional activities or tied to concrete research study tasks to enhance regular and long-term participation.

## Introduction

### Background

Electronic health diaries are consumer- or patient-facing electronic tools to record personal health-relevant information prospectively [1]. Flexibility and ease of use make electronic health diaries an attractive option for self-learning, patient empowerment, and health care management support [2]. Electronic health diaries are also used in research contexts where they offer more timely or even real-time reporting of health-related indicators and can thus meaningfully complement medical records or retrospective questionnaires [1]. In addition, free-text entries of diary studies can offer a window into a person’s emotions [3-6] and daily-life contexts.

Electronic health diaries have also found application in multiple sclerosis (MS) research, where they have been used as logbooks of physical activity sessions [7] or to record disability- or health-related quality of life scores [8-10]. Furthermore, health diaries have also been explored in the context of self-management improvement programs for persons with MS [11,12], as well as to monitor and enhance medication adherence to disease-modifying treatments (eg, through reminder functionalities) [13,14].

However, numerous methodological and user challenges have been highlighted [1,15]. First, keeping health diaries over extended periods (ie, months or even years) demands continual intrinsic and extrinsic motivation, as well as substantial support from study investigators [1,15]. Second, studies relying on free-text entries require special analytical methods [1,15] that involve substantial manual data preprocessing. Finally, to increase compliance, the number of diary questions needs to be relatively small—thus limiting the depth of health information that can realistically be collected (eg, regarding a person’s disease history).

### Objectives

In light of these challenges, we aimed to explore the applicability and usability of electronic health diary data collected by the Swiss Multiple Sclerosis Registry (SMSR) during a week-long health diary campaign. Specifically, we aimed to compare the characteristics of the participants in the health diary campaign with eligible nonparticipants of the SMSR campaign (aim 1). Furthermore, we intended to analyze the content of the diary free-text field section by applying 2 different natural language processing methods (aim 2). Finally, we aimed to evaluate the diary-collected health-related quality of life, symptoms, and treatment information by comparing it with corresponding survey-based information (aim 3).

These investigations were guided by several literature- and research-based assumptions. Recent research has revealed that younger age is linked to a more frequent adoption of mobile health technologies [1,2]. Therefore, we hypothesized that the electronic health diary campaign participants would be younger than nonparticipants with access to the registry’s web-based platform. Furthermore, although there is usually very good intraindividual consistency of stable symptoms, treatments, or side effects among semiannual surveys [16,17], the health diary may be better able to capture dynamic symptoms and treatment effects in a more comprehensive and fine-grained fashion.

## Methods

### Study Context

The electronic health diary data were collected by the SMSR as part of a nested campaign entitled “A week in the life of persons with MS,” which ran from March 9, 2019, to March 17, 2019. The SMSR is a nationwide, patient-centered registry for persons with MS (trial registration: ClinicalTrials.gov NCT02980640) [18,19]. The registry was launched in June 2016 as a collaboration between the Swiss Multiple Sclerosis Society and the University of Zurich with the aim to create a patient-centered registry for adult persons with MS living in Switzerland.

Since its launch in 2016, the SMSR has been collecting and updating standard information on MS symptoms, treatment histories, and health-related quality of life in semiannual surveys [19]. As of July 13, 2019, a total of 2350 participants had contributed data to the SMSR. Participants can either enroll for digital participation (approximately 80% of all participants), or they can choose to complete the surveys on paper (approximately 20%). The baseline assessment has 2 stages. First, participants complete a short survey (initial questionnaire) that collects basic sociodemographic characteristics and core data on MS diagnosis, as well as symptom and treatment histories. A second, more extensive survey (baseline questionnaire) collects current symptoms and treatments, as well as detailed information on education, work, health care use, comorbidities, and MS status [18,19].

The electronic health diary is a separate feature on the digital SMSR study platform, available in German, French, and Italian, and specifically developed to work on PCs and mobile devices. The diary was semistructured (Figures S1-S6 in Multimedia Appendix 1). It contained a free-text field where participants could describe their current health status and any other information that they deemed relevant. There were no restrictions on the number of characters. In addition, the diary included structured questions that mirrored corresponding survey items from the longitudinal surveys, namely the EQ-5D-5L and the EuroQol visual analog scale (EQ-VAS; 0-100 scale), as well as current MS symptoms (checkboxes) and current use of immunomodulatory and complementary therapies (checkboxes; Figures S5 and S6 in Multimedia Appendix 1). Visually, the diary was organized as a calendar sheet that allowed retrospective entries.

### Description of the Nested Electronic Health Diary Campaign

To promote the release of the electronic health diary, the SMSR launched a campaign entitled “A week in the life of persons with MS,” which ran from March 9, 2019, to March 17, 2019. All web-based participants were invited to make daily diary entries in the free-text field and complete the structured questions on current symptoms and medication intake (Multimedia Appendix 2). Participants were encouraged to share positive and negative experiences as well as self-management strategies.

The campaign included different communication activities (Multimedia Appendix 3, red numbers). First, a general announcement was issued by email on January 28, 2019. One month later (February 28, 2019), a personal invitation was sent by email to all web-based participants, followed by a reminder on March 8, 2019 (ie, the day before the launch of the campaign). A final motivational reminder was sent through email on March 14, 2019. An announcement and daily campaign updates were posted on the SMSR website as well as on the public website and the Facebook page of the Swiss Multiple Sclerosis Society. The daily campaign updates included visual summaries of different health diary items (average EQ-VAS score, percentage of participants with a specific mood, the 3 most frequent symptoms, percentage of users of complementary therapies, and the 3 most frequent complementary therapies).

### Study Population

**Figure 1:**
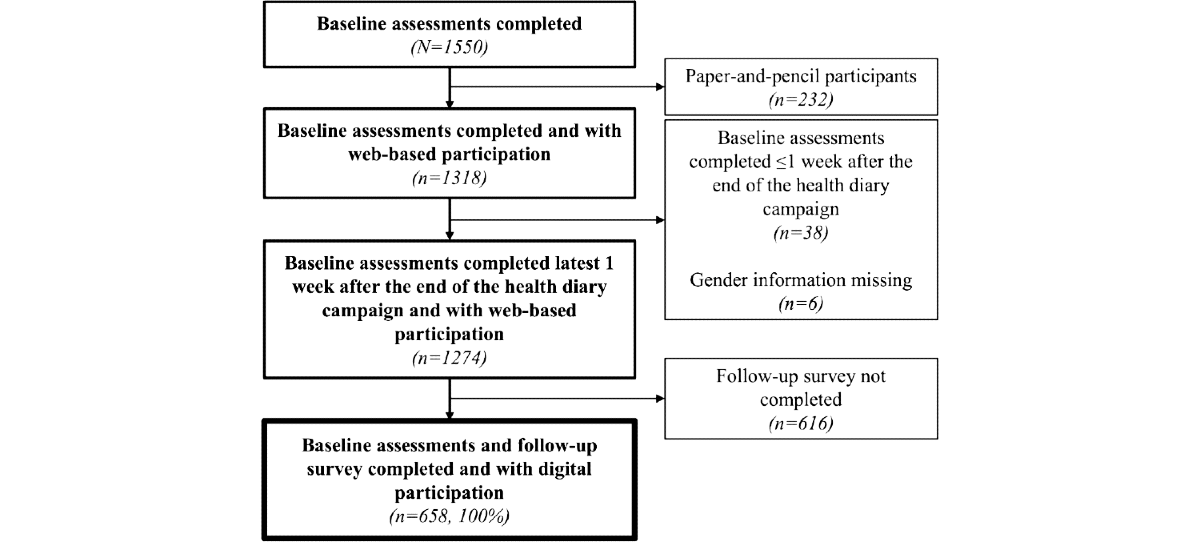
Flowchart of the study population, from the perspective of the completion of the baseline and follow-up surveys (refer to Multimedia Appendix 3 for more detailed information regarding the electronic health diary entries).

Of the 1550 SMSR enrollees who had completed the baseline assessments, 1318 (85.03%) participated through the SMSR web platform (Figure 1). Paper-and-pencil participants were excluded a priori because the health diary was only available on the web. After the application of inclusion criteria and data quality checks, we included 96.66% (1274/1318) of the web-based enrollees with baseline assessments completed until 1 week after the campaign ended (March 26, 2019). This time frame was chosen because a small number of participants (n=21) joined the SMSR during the diary campaign but completed the baseline assessment only a few days after the campaign ended. Of these 1274 enrollees, 658 (51.65%) had completed the next semiannual survey in the spring of 2020—hereafter *follow-up* survey—that included all standardized assessments from the diary (health-related quality of life, symptoms, and medication use). Among these 658 enrollees, 134 (20.4%) had made a nonempty free-text entry in the electronic health diary collected between February 27, 2019 (1 day before the invitation to the campaign was emailed), and March 19, 2019 (2 days after the official end of the campaign), including retrospective entries (Multimedia Appendix 4). This analysis time frame was chosen because the first campaign announcement on February 28, 2019, had already triggered numerous entries (Multimedia Appendix 3). Entries for retrospective dates were included if they were created during the analysis time frame.

### Measures

We analyzed 3 different types of data items that were collected both in the diary and the follow-up survey. The first data item consisted of textual diary entries describing notable daily events or current physical and mental well-being. Only German diary entries containing at least one word were considered for the analysis. The second analysis included the EQ-5D-5L index (derived from French reference value sets [20-22]) and the EQ-VAS. The third type of data pertained to current symptoms and the use of immunomodulatory medications [20,23].

In the diary and in the follow-up survey, participants were asked to indicate their current MS symptoms and the MS symptoms they had experienced within the last 12 months, respectively, from the following list: visual impairments, speech disorders, swallowing difficulties (dysphagia), weakness, signs of paralysis, fatigue, paresthesia (eg, deafness and tingling), dizziness, pain, gait disorder, vestibular disorders, bladder disorders (eg, bladder weakness), spasms (muscle cramps), convulsions and tics, tremor, intestinal disorders (eg, constipation), epileptic convulsions, sexual disorders, memory disorders, depression, concentration problems, problems with spatial orientation, affective lability or lack of control over emotions, or other unspecified symptoms.

Any current use of an immunomodulatory MS drug (diary and follow-up survey) and use of an immunomodulatory MS drug within the last 6 months (follow-up survey) were collected based on the following medication list: teriflunomide (Aubagio), interferon beta-1a (Avonex), interferon beta-1b (Betaferon), glatiramer acetate (Copaxone), cyclophosphamide (Endoxan), interferon beta-1b (Extavia), fingolimod (Gilenya), azathioprine (Imurek), alemtuzumab (Lemtrada), rituximab (MabThera), laquinimod (Nerventra), mitoxantrone (Novantron), ocrelizumab (Ocrevus), peginterferon beta-1a (Plegridy), interferon beta-1a (Rebif), cyclosporine (Sandimmun), tetracosactide (Synacthen), dimethyl fumarate (Tecfidera), natalizumab (Tysabri), and other unspecified medications.

### Statistical Analyses

Baseline characteristics of diary campaign participants (that is, persons with at least one valid health diary entry during the analysis time frame) and nonparticipants were compared descriptively (aim 1). Continuous data were analyzed by medians and IQRs and categorical information by frequency counts and percentages. Using visual plots, we analyzed the frequency and patterns of diary use over time, intending to learn more about the impact of reminders on participants’ behavior.

For aim 2, the content of diary text entries was analyzed using the Linguistic Inquiry and Word Count (LIWC) 2015 software (Pennebaker Conglomerates, Inc) [24,25] using a German dictionary [26]. On the basis of this dictionary, the LIWC program scores the texts according to 118 word attributes and categories by dividing the number of words belonging to a specific attribute or category by the number of words within a text entry to standardize for diary entry length [24]. The following seven categories related to health and personal experience were selected for further inspection: (1) health, (2) body, (3) family, (4) friends, (5) work, (6) positive emotions (eg, happy, good, and love), and (7) negative emotions (eg, sadness, anger, and fear). Using these 7 word categories, we calculated the percentage of entries containing at least one category-specific keyword across all diary entries. In addition, we analyzed an LIWC-integrated summary score for emotional tone by plotting its distribution across all diary entries in a histogram. The LIWC-generated *emotional tone* score represents the overall emotional tone of a text, ranging from 0 (negative) to 100 (positive). The score is computed based on the difference between dictionary-based positive and negative emotion scores, but details of this proprietary algorithm are not publicly available [24,27].

Furthermore, German diary text entries containing at least 10 words were analyzed descriptively by visualizing them as a word cloud of the 100 most frequent words in Python (version 3.7; Python Software Foundation) in the Spyder integrated development environment (version 4.1.5). The text entries were prepared for the visualization in several preprocessing steps. Stop words (ie, words without a specific meaning such as *and* or *the*) were removed using an open-source German stop word list [28]; numerals and punctuation marks were removed as well. The remaining words were filtered to retain only nouns, adjectives, and verbs. Words with the same meaning were replaced manually by 1 unique word (eg, the word *work* replaced the following words: occupation, job, company, office, workplace, work hours, work colleagues, etc). The words were then lemmatized using the Python library *spaCy* (version 2.2.2) [29]. Lemmatization refers to the removal of inflectional endings to obtain the dictionary form of a word [30]. Vowels followed by an *e* (such as *oe*) were replaced by a vowel with an umlaut (in this specific case, *ö*) as commonly used in German. Capital letters were then replaced by lowercase letters. Finally, all lemmatized entries were translated into English using DeepL Pro (DeepL SE).

Moreover, we evaluated the individual-level similarity of health-related quality of life indices (EQ-5D-5L and EQ-VAS), symptoms, and medication use between the structured diary data and follow-up survey data (aim 3). Similarity was expressed by the Jaccard index, which reflects the proportion of persons with the concurrent mention of a specific attribute value (eg, a symptom) in the diary and the follow-up survey (overlap), divided by the number of persons with at least one mention of the same attribute value in either the diary or the follow-up survey (union) [31,32]. The Jaccard index was chosen over standard percentages because it is less affected by the rarity of an attribute value. In case a participant had several complete health diary entries, the health-related quality of life indices of the closest follow-up survey were used for the similarity analyses. The diary-based categorical symptom and medication use variables were compared with similar follow-up survey information on symptoms and medication use. The continuous EQ-5D-5L and EQ-VAS scores were dichotomized using median splits to calculate the Jaccard indices. The respective medians were computed from the follow-up survey of all included SMSR participants (n=658).

All descriptive analyses were conducted in R (version 4.0.3), using the RStudio integrated development environment (version 1.4.1103). Text preprocessing and word cloud visualization were performed using Python (version 3.7) in the Spyder integrated development environment (version 4.1.5). The dictionary-based text categorizations were performed using the LIWC software [24,25]. All text analyses were conducted using the original German text entries, and the results were subsequently translated into English for presentation using DeepL Pro.

### Ethics Approval

The SMSR has been approved by the responsible ethics committee (Zurich Cantonal Ethics Committee; study number PB-2016-00894), and informed consent was obtained from all participants.

## Results

### Study Populations and Diary Use

After we had applied our inclusion and exclusion criteria, our study population comprised 658 persons with MS, of whom 134 (20.4%) had used the electronic health diary between February 27, 2019, and March 19, 2019 (Figure 1). During this period, 815 nonempty, unique diary entries written in German (n=632, 77.5%), French (n=135, 16.6%), and Italian (n=48, 5.9%) were collected (Multimedia Appendix 4).

The median time span between the last diary entry written by each diary campaign participant and the follow-up survey was 14 (IQR 13-15) months.

### Participant Characteristics

Characteristics of the SMSR web-based study enrollees who participated in the campaign by completing at least one electronic health diary entry (n=134; henceforth referred to as *participants*) and those who did not (n=524; henceforth referred to as *nonparticipants*) are compared in Table 1. The median age of the participants (52.5, IQR 43.25-59.75 years) was higher than that of the nonparticipants (47, IQR 38-55 years). Of the 134 participants, 101 (75.4%) were women, whereas of the 524 nonparticipants, 356 (67.9%) were women. The median time since the diagnosis of MS was 9 (IQR 4-19 for participants and IQR 4-16 for nonparticipants) years for both groups. Persons with primary progressive MS (PPMS) and secondary progressive MS (SPMS) tended to be more represented among participants (PPMS: 18/134, 13.4%; SPMS: 32/134, 23.9%) than among nonparticipants (PPMS: 50/524, 9.5%; SPMS: 79/524, 15.1%). In addition, the participants’ group contained proportionally fewer persons in the lowest Self-Reported Disability Status Scale stratum (84/134, 62.7%) than the nonparticipants’ group (381/524, 72.7%).

The nonparticipants’ group comprised a somewhat larger percentage of persons with higher professional education (including with an applied university or university degree; 250/524, 47.7%, vs 57/134, 42.5%, for participants). However, there were fewer people working >40% of weekly working hours among the participants (45/134, 33.6%, vs 252/524, 48.1%, for nonparticipants). Nonparticipants seemed to talk more openly about their MS with their relatives (485/524, 92.5%, vs 116/134, 86.6%, for participants).

Regarding symptoms experienced within the last 12 months, participants reported a higher percentage for the 10 most frequent symptoms. The largest differences in percentages were observed for the following symptoms: gait disorder (52/134, 38.8%, vs 138/524, 26.3%, for nonparticipants), spasms (45/134, 33.6%, vs 119/524, 22.7%, for nonparticipants), and bladder disorders (40/134, 29.9%, vs 109/524, 20.8%, for nonparticipants). Furthermore, a higher proportion of diary campaign participants reported no use of a disease-modifying drug within the last 6 months (53/134, 39.6%, vs 156/524, 29.8%, for nonparticipants). The full list of symptoms and disease-modifying medications is available in Multimedia Appendix 5.

**Table 1:** Characteristics of the study population and their corresponding *P* values (significance level: .05; N=658).^a^

Characteristics		Participants in health diary campaign (n=134)	Nonparticipants (n=524)	*P* value
Age (years), median (IQR)		52.5 (43.25-59.75)	47 (38-55)	<.001^b^
**Sex, n (%)**				.12^c^
	Female	101 (75.4)	356 (67.9)	
	Male	33 (24.6)	168 (32.1)	
**Language, n (%)**				<.001^c^
	German	100 (74.6)	433 (82.6)	
	French	25 (18.7)	73 (13.9)	
	Italian	9 (6.7)	18 (3.5)	
MS^d^ duration (years), median (IQR)		9 (4-19)	9 (4-16)	.17^b^
**MS type, n (%)**				<.001^c^
	CIS^e^	2 (1.5)	10 (1.9)	
	PPMS^f^	18 (13.4)	50 (9.5)	
	RRMS^g^	79 (59)	352 (67.2)	
	Transition	3 (2.2)	16 (3.1)	
	SPMS^h^	32 (23.9)	79 (15.1)	
	Missing information	N/A^i^	17 (3.2)	
**Marital status, n (%)**				<.001^c^
	Unmarried	43 (32.1)	174 (33.2)	
	Registered partnership or married	71 (53)	273 (52.1)	
	Separated or divorced	17 (12.7)	59 (11.3)	
	Widowed	1 (0.7)	8 (1.5)	
	Missing information	2 (1.5)	10 (1.9)	
**Education, n (%)**				.59^c^
	Partial or completed mandatory schooling	3 (2.2)	8 (1.5)	
	Apprenticeship or qualification to study at university level (Matura diploma)	68 (50.8)	250 (47.7)	
	Higher professional education, applied university, or university	57 (42.5)	250 (47.7)	
	Other	1 (0.8)	7 (1.4)	
	Missing information	5 (3.7)	9 (1.7)	
**Work situation or work percentage, n (%)**				.02^c^
	Not working	63 (47)	184 (35.1)	
	1% to 40%	22 (16.4)	69 (13.2)	
	41% to 80%	27 (20.2)	138 (26.3)	
	81% to full time	18 (13.4)	114 (21.8)	
	Missing information	4 (3)	19 (3.6)	
**Talk about MS with...^j^**				.87^c^
	...relatives	116 (86.6)	485 (92.6)	
	...friends	109 (81.3)	412 (78.6)	
	...boss	48 (35.8)	179 (34.2)	
	...work colleagues	46 (34.3)	189 (36.1)	
	...leisure-time partners	36 (26.9)	131 (25)	
	...other	7 (5.2)	23 (4.4)	
	...nobody	6 (4.5)	13 (2.5)	
**SRDSS^k^ score, n (%)**				.02^c^
	0 to 3.5	84 (62.7)	381 (72.7)	
	4 to 6.5	37 (27.6)	90 (17.2)	
	≥7	13 (9.7)	39 (7.4)	
	Missing information	N/A	14 (2.7)	
**Ten most frequent symptoms within the last 12 months^j,l^, n (%)**				.42^c^
	None	50 (37.3)	227 (43.3)	
	Fatigue	57 (42.5)	210 (40.1)	
	Gait disorder	52 (38.8)	138 (26.3)	
	Paresthesia (eg, numbness and tingling)	50 (37.3)	187 (35.7)	
	Spasms (muscle cramps)	45 (33.6)	119 (22.7)	
	Vestibular disorders	43 (32.1)	135 (25.8)	
	Weakness	40 (29.9)	129 (24.6)	
	Pain	40 (29.9)	143 (27.3)	
	Bladder disorders (eg, bladder weakness)	40 (29.9)	109 (20.8)	
	Concentration problems	35 (26.1)	127 (24.2)	
	Intestinal disorders (eg, constipation)	29 (21.6)	83 (15.8)	
	Other	64 (47.8)	193 (36.8)	
**Ten most frequent disease-modifying medications within the last 6 months^j,l^, n (%)**				.20^c^
	None	53 (39.6)	156 (29.8)	
	Ocrelizumab (Ocrevus)	28 (20.9)	87 (16.6)	
	Fingolimod (Gilenya)	15 (11.2)	90 (17.2)	
	Dimethyl fumarate (Tecfidera)	10 (7.5)	54 (10.3)	
	Interferon beta-1a (Rebif)	6 (4.5)	19 (3.6)	
	Glatiramer acetate (Copaxone)	6 (4.5)	18 (3.4)	
	Interferon beta-1b (Betaferon)	5 (3.7)	14 (2.7)	
	Teriflunomide (Aubagio)	5 (3.7)	15 (2.9)	
	Natalizumab (Tysabri)	3 (2.2)	40 (7.6)	
	Rituximab (MabThera)	3 (2.2)	15 (2.9)	
	Interferon beta-1a (Avonex)	2 (1.5)	10 (1.9)	
	Other	1 (0.7)	10 (1.9)	

^a^Comparison between participants in the Swiss Multiple Sclerosis Registry electronic health diary campaign (defined by having at least one valid diary entry; column 2) and nonparticipants (column 3).

^b^Two-tailed *t* test conducted.

^c^Chi-square test conducted.

^d^MS: multiple sclerosis.

^e^CIS: clinically isolated syndrome.

^f^PPMS: primary progressive multiple sclerosis.

^g^RRMS: relapsing-remitting multiple sclerosis.

^h^SPMS: secondary progressive multiple sclerosis.

^i^N/A: not applicable.

^j^Multiple answers possible.

^k^SRDSS: Self-Reported Disability Status Scale.

^l^Full list of symptoms and disease-modifying medications available in Multimedia Appendix 5.

### Health Diary Use Patterns

The use of the electronic health diary was monitored over 21 days between February 27, 2019, and March 19, 2019 (Multimedia Appendix 3). Over these 21 days, a total of 815 unique electronic health diary entries were collected from 134 diary campaign participants. Of note, more health diary entries were collected on days when email invitations or reminders were sent than on the adjacent days; for example, the final reminder sent on March 14, 2019 (bar marked by a red numeral 3 in Multimedia Appendix 3), displayed the largest number of entries (n=130) of the whole observation period. In addition, the electronic health diary was used more intensively during the official health diary campaign period (striped bars in Multimedia Appendix 3).

As shown in Table 2, 23.1% (31/134) of the participants wrote only 1 entry in the diary, and 11.2% (15/134) wrote 2. Moreover, 15.7% (21/134) of the participants had ≥10 entries (reflecting ≥1 entry every 2 days). The highest number of entries written by 1 person was 32 (including retrospective entries made during the analysis time frame). Multimedia Appendix 6 summarizes the completeness of health diary entries.

**Table 2:** Frequency counts and percentages of participants totalizing a certain number of diary health entries written during the campaign (N=134).

Number of entries in the health diary per participant	Participants, n (%)
1	31 (23.1)
2	15 (11.2)
3	6 (4.5)
4	5 (3.7)
5	6 (4.5)
6	6 (4.5)
7	9 (6.7)
8	16 (11.9)
9	19 (14.2)
10	6 (4.5)
11	5 (3.7)
12	2 (1.5)
13	1 (0.7)
15	2 (1.5)
17	1 (0.7)
18	1 (0.7)
19	1 (0.7)
20	1 (0.7)
32	1 (0.7)

### Content Analysis of the Textual Diary Data

The 134 electronic health diary campaign participants provided a total of 815 electronic health diary entries (in German: n=632, 77.5%; in French: n=135, 16.6%; and in Italian: n=48, 5.9%). For the subsequent analysis, we only used the German health diary entries, provided by 100 participants. The emotional scores of the 632 diary entries were low overall, reflecting negative emotions such as very anxious, sad, or hostile tone [33]. Of the 632 diary entries, 163 (25.8%) scored between 0 and 10, and 420 (66.5%) scored between 20 and 30. Furthermore, positive emotions were expressed in only 7.6% (48/632) of the entries, whereas words related to negative emotions were used in 30.5% (193/632) of the entries (Table 3). Other frequent LIWC-based topic categories pertained to the body (191/632, 30.2%), health (94/632, 14.9%), and work (67/632, 10.6%).

Of the 632 German electronic health diary entries, 526 (83.2%; written by 93 participants) contained at least 10 words and were used to create a 100-word word cloud (Multimedia Appendix 7; refer to Multimedia Appendix 8 for the frequency of the 25 most common words). As shown in Multimedia Appendix 7 and Multimedia Appendix 8, the words “good” and “day” occur most often, followed by “work,” “up,” “go,” “sleep,” and “tired.” Among the 25 most frequent words, some refer to time, including “day,” “evening,” “morning,” “hour,” “time,” and “afternoon.” Other words pertained to activities or movement, such as “work,” “go,” “walk,” “make,” and “errand.” Several frequent words also related to body parts, physical well-being, and health issues; for example, “tired,” “leg,” “pain,” “therapy,” and “feel.” Although comparatively fewer words were associated with social contacts, some were very common in diary entries, such as “contact” or “family.” A word cloud and the frequency of the most common words for each MS type (ie, PPMS, relapsing-remitting multiple sclerosis, transition, and SPMS) are available in Multimedia Appendix 9.

**Table 3:** Frequency counts and percentages of the diary entries containing relevant Linguistic Inquiry and Word Count word categories (N=632).

Characteristics	Values, n (%)
Negative emotion	193 (30.5)
Body	191 (30.2)
Health	94 (14.9)
Work	67 (10.6)
Positive emotion	48 (7.6)
Friends	10 (1.6)
Family	8 (1.3)

### Similarities Between Structured Electronic Health Diary Data and Questionnaire-Based Data

The responses to the electronic health diary and the follow-up survey exhibited several notable differences and similarities quantified by the Jaccard index (Table 4). The EQ-5D-5L (Jaccard indices: 0.73 and 0.63 below and above the median, respectively) and the EQ-VAS (Jaccard indices: 0.59 and 0.49, respectively) presented the highest Jaccard indices. Furthermore, the electronic health diary and the follow-up survey methods converged for the evaluation of gait disorder, signs of paralysis, and fatigue (Jaccard indices: 0.5, 0.5, and 0.49, respectively). By contrast, several symptoms, including pain, vestibular disorder, weakness, spasms, bladder disorders, and concentration problems displayed comparatively lower Jaccard indices (<0.4), despite being reported quite frequently (n>45; refer to the column *Union* in Table 4 and the *Ten most frequent symptoms within the last 12 months* section in Table 1) and at least once in the diary and the survey. The injectable immunomodulatory drugs interferon beta-1a (Avonex) and glatiramer acetate (Copaxone), as well as the oral drugs fingolimod (Gilenya) and teriflunomide (Aubagio), exhibited the highest similarities across both assessment methods (Jaccard indices between 0.5 and 1; refer to Multimedia Appendix 10 for the complete list of disease-modifying medications). Of note, despite currently being used frequently, ocrelizumab (Ocrevus) was not mentioned in the diary.

**Table 4:** Similarity between diary and follow-up survey information on health-related quality of life, symptoms, and medication use provided by 134 electronic health diary campaign participants.^a^

Characteristics		Overlap	Union	Jaccard index
**EQ-5D-5L**				
	EQ-5D-5L: ≤0.71 (overall median)	52	71	0.73
	EQ-5D-5L: >0.71	33	52	0.63
**EQ-VAS^b^**				
	EQ-VAS: ≤80 (overall median)	34	58	0.59
	EQ-VAS: >80	23	47	0.49
**Symptoms**				
	Gait disorder	33	66	0.50
	Signs of paralysis	12	24	0.50
	Fatigue	39	80	0.49
	Paresthesia (eg, numbness and tingling)	34	73	0.47
	Tremor	8	18	0.44
	Intestinal disorders (eg, constipation)	14	34	0.41
	Weakness	22	58	0.38
	Vestibular disorders	23	62	0.37
	Pain	22	63	0.35
	Affective lability and lack of control over emotions	5	15	0.33
	Spasms (muscle cramps)	18	56	0.32
	Memory disorders	9	28	0.32
	Bladder disorders (eg, bladder weakness)	16	51	0.31
	Convulsions and tics	5	17	0.29
	Sexual disorders	5	19	0.26
	Visual impairments	7	30	0.23
	Problems with spatial orientation	7	30	0.23
	Dizziness	7	32	0.22
	Speech disorders	4	20	0.20
	Swallowing difficulties (dysphagia)	3	15	0.20
	Depression	3	16	0.19
	Concentration problems	7	46	0.15
	Other	0	10	0
	Epileptic convulsions	0	1	0
**Ten most frequent disease-modifying medications ^c^**				
	Interferon beta-1a (Avonex)	2	2	1
	Glatiramer acetate (Copaxone)	5	7	0.71
	Fingolimod (Gilenya)	12	21	0.57
	Teriflunomide (Aubagio)	4	8	0.50
	Interferon beta-1b (Betaferon)	2	5	0.40
	Dimethyl fumarate (Tecfidera)	1	32	0.03
	Ocrelizumab (Ocrevus)	0	36	0
	Other	0	33	0
	Interferon beta-1a (Rebif)	0	6	0
	Natalizumab (Tysabri)	0	6	0
	Rituximab (MabThera)	0	3	0

^a^The Jaccard index was used to measure the similarities (reporting overlap) among the different sources (0=no similarity and 1=maximum similarity). The column *Overlap* represents the number of participants with overlapping reports in the diary and in the follow-up survey. The column *Union* represents the total number of participants who reported a specific data item at least once in the diary or the follow-up survey collecting the symptoms experienced within the last 12 months and the medication used within the last 6 months. The column *Jaccard index* represents the quotient of the *Overlap* values and the *Union* values. The EQ-5D-5L index and the EuroQol visual analog scale index were dichotomized using the respective medians of all included Swiss Multiple Sclerosis Registry enrollees with completed follow-up survey (n=658).

^b^EQ-VAS: EuroQol visual analog scale.

^c^The full list of disease-modifying medications is available in Multimedia Appendix 10.

## Discussion

### Principal Findings

On the basis of a health diary campaign nested into the SMSR, our study sought to explore the factors associated with electronic health diary use as well as the added value of diary entries vis-à-vis survey-collected information. By comparing the characteristics of 134 health diary campaign participants with those of 524 nonparticipants, our study revealed that women, persons with a more advanced disease state, those with a lower work percentage, or those without higher education (eg, a university degree) were more likely to devote time to the electronic health diary.

Contrary to our initial expectations, electronic health diary participants were on average 5 years older than the nonparticipants, more likely to be women, less likely to have completed higher education, and more frequently had an advanced Self-Reported Disability Status Scale score (≥4). In addition, a larger proportion of participants reported experiencing diverse MS symptoms within the last 12 months, in particular from more strongly hindering symptoms such as fatigue, gait disorder, spasms, and vestibular disorders. Not only are these symptoms disabling, but they may also lead to a more confined life at home. These findings may seem counterintuitive at first glance because studies comparing characteristics between web-based and paper-and-pencil study participants (including a study from the SMSR) usually found younger age, higher education, and less disease severity to be associated with a higher probability of web-based participation [34,35]. However, this study focused entirely on web-based participants. Thus, the stratification by health diary use reflects a further segmentation of this population. In our analysis, persons with a higher symptom burden and not working full time, which are two factors associated with older age, were more likely to contribute to the electronic health diary campaign. This finding overlaps with an independent SMSR analysis showing that, based on a set of standardized questions, persons with a high affinity for using digital tools for MS management also tended to be older (but still middle-aged) and to have a higher disease burden (Nittas, unpublished data, March 2022). It can be speculated that an elevated disease burden seems to come with more time at disposal and a stronger desire to tell one’s story as well as to understand one’s disease.

When analyzing the content of health diary entries, participants’ reports mainly addressed themes related to the body, health, and work, as confirmed by the LIWC and word cloud analytic approaches. Moreover, the frequent mention of words such as “sleep” or “tired” corresponds well with frequent reports of fatigue in the diary as well as in the SMSR follow-up survey (Table 1).

A substantial number of participant reports had a negative emotional coloration and were referring to, for example, the adverse effects of MS on their body and daily life activities. Indeed, keywords from the LIWC categories *negative emotion* and *body* appeared in the same proportion of diary entries and were thus possibly co-occurring. Diary participants seemed to rather express concerns—possibly about their disease affecting their body and their active life. Whereas a larger proportion of the nonparticipants talk about MS to their relatives, diary participants seemed to confide their worries in the diary [36].

Of further note, the comparison of structured information from the electronic health diary and the follow-up survey generally exhibited good overlaps for health-related quality of life aspects, relatively stable and frequent symptoms, and—to a lesser extent—for medications with a frequent intake schedule (eg, injectable or oral drugs). The health-related quality of life index EQ-5D-5L revealed the highest overlap, which is consistent with the general notion that this indicator is not very responsive to smaller health status changes. The more subjective EQ-VAS showed somewhat smaller overlaps, possibly because the EQ-VAS is more responsive to daily fluctuations [9]. The Jaccard index was also relatively high for stable or slow-changing symptoms such as gait disorder, signs of paralysis, and fatigue. By contrast, the similarity analysis of medication use mostly yielded low Jaccard indices, suggesting little overlap. Nonetheless, older, established injection therapies for relapsing-remitting MS, such as interferon beta-1a (Avonex) [37] and glatiramer acetate (Copaxone) [38], although not used by a large proportion of our study participants, displayed the highest Jaccard indices, demonstrating little change in medication. Furthermore, more modern and convenient treatments such as fingolimod (Gilenya) and teriflunomide (Aubagio), which are two oral therapies with daily intake for relapsing-remitting MS, also exhibited relatively high Jaccard indices. Nonetheless, other modern MS drugs administered in monthly (natalizumab [Tysabri]) infusions at clinics demonstrated low Jaccard indices because of the diary instructions that asked participants for MS medication in the previous day only. Furthermore, the frequently used infusion drug ocrelizumab (Ocrevus) was only widely prescribed in Switzerland after the health diary campaign ended [39].

To the best of our knowledge, this is the first study to assess the similarities between data collected through a web-based survey and data collected by means of an electronic diary. The different person and disease characteristics associated with electronic health diary use can help refine the target population for implementing electronic health diary tools in research contexts. Furthermore, the findings suggest that health diaries can form a meaningful complement to regular surveys by providing additional insights into daily life topics and timely updates on health-related quality of life or symptom and medication status of persons with MS. Although ecological momentary assessment research has highlighted the possible risk of retrospection bias in retrospectively reporting symptoms averaged over longer times [40], subjective perceptions of illnesses vary strongly and meaningfully across people [41]. Indeed, evaluating subjective views with regard to, for example, symptoms of people with MS not only adds value to standardized assessments pertaining to personalized treatment planning but also helps to explain adherence to treatment regimens [42]. Moreover, free-text questions as implemented in our diary can complement regular standardized assessments by providing additional levels of detail. Furthermore, being given the opportunity to report on their experiences in their own words is often appreciated by study participants and, as suggested by the extensive expressive writing literature, may even be of therapeutical value in itself [43]. Our electronic health diary combines the known advantages of ecological momentary assessment with the advantage of not restricting answers to forced, given choices.

The study was also informative on a methodological level. Although the health diary has been part of the SMSR platform since its launch, voluntary use was very low, on average <1 entry per day per participant. By contrast, we noticed that the use of the electronic health diary was enhanced after motivational emails were sent to the participants. These experiences suggest that health diary studies may benefit from being embedded into a campaign with clear aims and a limited time frame, as revealed by previous studies [44,45].

### Strengths and Limitations

Our study benefited from a large, diverse, well-documented study base for enrollment [18,19]. Moreover, this is the first health diary study in MS to use a blend of different analytical approaches (descriptive statistics and natural language processing) to glean insights into health diary use patterns and daily life aspects; for example, closer inspection of diary entries revealed novel aspects such as the stress imposed by application procedures for disability insurance or individual coping strategies for well-being by persons with MS (not shown). Therefore, diary studies harbor a significant untapped potential for hypothesis generation and inspiration for research topics.

However, some limitations should be noted. First, our study excluded persons who preferred paper-and-pencil surveys. This excluded group reflects a population with more advanced disease states and possibly lower digital literacy (Nittas, unpublished data, March 2022). However, similar to our study, earlier investigations also observed that at an early stage of the disease, persons with MS are less likely to engage in research studies (Nittas, unpublished data, March 2022), [46], hypothetically because they are in denial regarding their disease or do not entirely realize the scope of it yet. In addition, the distance in time between the health diary campaign and the follow-up survey completion was relatively long. Hence, the MS symptomatology, as well as treatment strategies, may have changed in the meantime (eg, ocrelizumab [Ocrevus] has started to be widely prescribed in Switzerland after the end of the health diary campaign). It is important to mention that the results could be replicated using different comparison data provided by the SMSR. Future research would benefit from further investigating the added value of such free-text health diaries by comparing entries with standardized instruments of physical and mental well-being assessed in parallel. Furthermore, among the 658 eligible web-based SMSR participants, only a fraction (n=134, 20.4%) took part in the diary study, thus limiting the generalizability of the results. Besides, the vast majority (113/134, 84.3%) of the participants made <1 entry every 2 days in the diary, and 34% (46/134) contributed only once or twice to the diary campaign. This lack of regularity in diary use was also a limitation to our study. The implementation of a diary with automated reminders [47] might be a promising way forward to increase participation [12,14]. Nevertheless, these limitations observed in our study may also hinder a broader application of electronic diaries in ecological momentary assessments.

Therefore, a better understanding of participant needs, motivational factors, and the effectiveness of incentives is urgently needed to enable a broader application and long-term use of health diaries in health research and disease management. In addition, in light of recent advances in fields such as natural language processing [48] and speech recognition [49] (eg, Hugging Face [50]) based on machine learning or wearable sensor technologies (eg, fitness trackers) [42,51,52], future studies should examine how health diaries could be optimally combined with novel technologies.

### Conclusions

To summarize, our study suggests that health diaries can be a valuable complement to regular, structured questionnaires in the context of MS research. However, they should ideally be embedded into a campaign with motivational activities such as email reminders or regular data feedback. Our findings further suggest that a topical focus on daily life aspects, health-related quality of life, and stable symptoms elicited more similar responses to standardized assessments and were thus less informative than medication diaries. Hence, medication diaries for daily dispensed (oral or injectable) drugs may offer opportunities for drug intake compliance tracking and unwanted drug effect occurrence monitoring.

## Appendix

Multimedia Appendix 1 Electronic health diary of the Swiss Multiple Sclerosis Registry.

Multimedia Appendix 2 Invitation letters to the electronic health diary campaign for the Swiss Multiple Sclerosis Registry participants.

Multimedia Appendix 3 Number of entries made per day in the electronic health diary between February 27, 2019, and March 19, 2019 (N=815). The striped bars (March 9, 2019, to March 17, 2019) correspond to the official health diary campaign period. The red numbers indicate when the Swiss Multiple Sclerosis Society sent emails to its participants: (1) invitation email for the campaign sent out to all Swiss Multiple Sclerosis Registry participants and information released as a news item on the public web page of the Swiss Multiple Sclerosis Society, (2) reminder email sent out to all participants informing them that the campaign was about to start, and (3) motivational email that provided some statistical information about the health diary campaign and reminded participants to contribute.

Multimedia Appendix 4 Flowchart of the Swiss Multiple Sclerosis Registry enrollees and the electronic health diary participants.

Multimedia Appendix 5 Full list of the symptoms experienced within the last 12 months and the disease-modifying medications used within the last 6 months.

Multimedia Appendix 6 Completeness of the diary entries.

Multimedia Appendix 7 Word cloud of the 100 most frequent words appearing in the electronic health diary entries of a minimum length of 10 words (n=526). The font size reflects the frequency of the words’ occurrence.

Multimedia Appendix 8 Word frequency of the 25 most frequent words of the word cloud.

Multimedia Appendix 9 Word cloud of 100 words and word frequency of the 25 most frequent words throughout the electronic health diary entries per multiple sclerosis type.

Multimedia Appendix 10 Jaccard indices of the full disease-modifying medication list.

## Abbreviations

EQ-VAS: EuroQol visual analog scale
LIWC: Linguistic Inquiry and Word Count
MS: multiple sclerosis
PPMS: primary progressive multiple sclerosis
SMSR: Swiss Multiple Sclerosis Registry
SPMS: secondary progressive multiple sclerosis

## References

[1] Verbrugge LM (1980). Health diaries. Med Care.

[2] Al-Ubaydli M, Paton C (2005). The doctor's PDA and smartphone handbook. Personal digital assistant. J R Soc Med.

[3] Pennebaker JW (1993). Putting stress into words: health, linguistic, and therapeutic implications. Behav Res Ther.

[4] Pennebaker JW, Barger SD, Tiebout J (1989). Disclosure of traumas and health among Holocaust survivors. Psychosom Med.

[5] Smyth JM (1998). Written emotional expression: effect sizes, outcome types, and moderating variables. J Consult Clin Psychol.

[6] Frattaroli J (2006). Experimental disclosure and its moderators: a meta-analysis. Psychol Bull.

[7] Sebastião E, McAuley E, Shigematsu R, Adamson BC, Bollaert RE, Motl RW (2018). Home-based, square-stepping exercise program among older adults with multiple sclerosis: results of a feasibility randomized controlled study. Contemp Clin Trials.

[8] Freeman J, Hendrie W, Jarrett L, Hawton A, Barton A, Dennett R, Jones B, Zajicek J, Creanor S (2019). Assessment of a home-based standing frame programme in people with progressive multiple sclerosis (SUMS): a pragmatic, multi-centre, randomised, controlled trial and cost-effectiveness analysis. Lancet Neurol.

[9] Parkin D, Rice N, Jacoby A, Doughty J (2004). Use of a visual analogue scale in a daily patient diary: modelling cross-sectional time-series data on health-related quality of life. Soc Sci Med.

[10] Bove R, Secor E, Healy BC, Musallam A, Vaughan T, Glanz BI, Greeke E, Weiner HL, Chitnis T, Wicks P, De Jager PL (2013). Evaluation of an online platform for multiple sclerosis research: patient description, validation of severity scale, and exploration of BMI effects on disease course. PLoS One.

[11] Jongen PJ, Sinnige LG, van Geel BM, Verheul F, Verhagen WI, van der Kruijk RA, Haverkamp R, Schrijver HM, Baart JC, Visser LH, Arnoldus EP, Gilhuis HJ, Pop P, Booy M, Lemmens W, Donders R, Kool A, van Noort E (2015). The interactive web-based program MSmonitor for self-management and multidisciplinary care in multiple sclerosis: concept, content, and pilot results. Patient Prefer Adherence.

[12] Golan D, Sagiv S, Glass-Marmor L, Miller A (2021). Mobile-phone-based e-diary derived patient reported outcomes: association with clinical disease activity, psychological status and quality of life of patients with multiple sclerosis. PLoS One.

[13] Jongen PJ, Sinnige LG, van Geel BM, Verheul F, Verhagen WI, van der Kruijk RA, Haverkamp R, Schrijver HM, Baart JC, Visser LH, Arnoldus EP, Gilhuis HJ, Pop P, Booy M, Heerings M, Kool A, van Noort E (2016). The interactive web-based program MSmonitor for self-management and multidisciplinary care in multiple sclerosis: utilization and valuation by patients. Patient Prefer Adherence.

[14] Zettl UK, Bauer-Steinhusen U, Glaser T, Czekalla J, Hechenbichler K, Limmroth V, Hecker M (2016). Adherence to long-term interferon beta-1b injection therapy in patients with multiple sclerosis using an electronic diary. Adv Ther.

[15] Richardson A (1994). The health diary: an examination of its use as a data collection method. J Adv Nurs.

[16] Gajofatto A, Benedetti MD (2015). Treatment strategies for multiple sclerosis: when to start, when to change, when to stop?. World J Clin Cases.

[17] Ciotti JR, Cross AH (2018). Disease-modifying treatment in progressive multiple sclerosis. Curr Treat Options Neurol.

[18] Puhan MA, Steinemann N, Kamm CP, Müller S, Kuhle J, Kurmann R, Calabrese P, Kesselring J, von Wyl V, Swiss Multiple Sclerosis Registry Smsr (2018). A digitally facilitated citizen-science driven approach accelerates participant recruitment and increases study population diversity. Swiss Med Wkly.

[19] Steinemann N, Kuhle J, Calabrese P, Kesselring J, Disanto G, Merkler D, Pot C, Ajdacic-Gross V, Rodgers S, Puhan MA, von Wyl V, Swiss Multiple Sclerosis Registry (2018). The Swiss Multiple Sclerosis Registry (SMSR): study protocol of a participatory, nationwide registry to promote epidemiological and patient-centered MS research. BMC Neurol.

[20] Barin L, Salmen A, Disanto G, Babačić H, Calabrese P, Chan A, Kamm CP, Kesselring J, Kuhle J, Gobbi C, Pot C, Puhan MA, von Wyl V, Swiss Multiple Sclerosis Registry (SMSR) (2018). The disease burden of Multiple Sclerosis from the individual and population perspective: which symptoms matter most?. Mult Scler Relat Disord.

[21] Matter-Walstra K, Klingbiel D, Szucs T, Pestalozzi BC, Schwenkglenks M (2014). Using the EuroQol EQ-5D in Swiss cancer patients, which value set should be applied?. Pharmacoeconomics.

[22] Perneger TV, Combescure C, Courvoisier DS (2010). General population reference values for the French version of the EuroQol EQ-5D health utility instrument. Value Health.

[23] McGinley MP, Goldschmidt CH, Rae-Grant AD (2021). Diagnosis and treatment of multiple sclerosis: a review. JAMA.

[24] Pennebaker J, Boyd R, Jordan K, Blackburn K (2015). The development and psychometric properties of LIWC2015. University of Texas.

[25] Welcome to LIWC-22. LIWC.

[26] Meier T, Boyd R, Pennebaker J, Mehl M, Martin M, Wolf M, Horn A (2019). “LIWC auf Deutsch”: the development, psychometrics, and introduction of DE- LIWC2015. PsyArXiv.

[27] Cohn MA, Mehl MR, Pennebaker JW (2004). Linguistic markers of psychological change surrounding September 11, 2001. Psychol Sci.

[28] Diaz G (2020). GitHub - stopwords-iso/stopwords-de: German stopwords collection. GitHub.

[29] Honnibal M, Montani I (2017). spaCy 2: natural language understanding with Bloom embeddings, convolutional neural networks and incremental parsing. Spacy.

[30] Ahn I, Na W, Kwon O, Yang DH, Park G, Gwon H, Kang HJ, Jeong YU, Yoo J, Kim Y, Jun TJ, Kim Y (2021). CardioNet: a manually curated database for artificial intelligence-based research on cardiovascular diseases. BMC Med Inform Decis Mak.

[31] Jaccard P (1912). The distribution of the flora in the alpine zone. New Phytol.

[32] Rhee S, Liu TF, Holmes SP, Shafer RW (2007). HIV-1 subtype B protease and reverse transcriptase amino acid covariation. PLoS Comput Biol.

[33] Pennebaker J, Booth R, Boyd R, Francis M (2015). Linguistic inquiry and word count: LIWC2015. Pennebaker Conglomerates.

[34] Potdar R, Thomas A, DiMeglio M, Mohiuddin K, Djibo DA, Laudanski K, Dourado CM, Leighton JC, Ford JG (2020). Access to internet, smartphone usage, and acceptability of mobile health technology among cancer patients. Support Care Cancer.

[35] Fox G, Connolly R (2018). Mobile health technology adoption across generations: narrowing the digital divide. Info Systems J.

[36] Grose J, Freeman J, Skirton H (2012). Value of a confidant relationship in psychosocial care of people with multiple sclerosis. Int J MS Care.

[37] Why Avonex. Avonex.

[38] Wynn DR (2019). Enduring clinical value of Copaxone® (Glatiramer Acetate) in multiple sclerosis after 20 years of use. Mult Scler Int.

[39] (2017). Roche’s OCREVUS (ocrelizumab) approved in Switzerland for primary progressive and relapsing forms of multiple sclerosis. Roche.

[40] Shiffman S, Stone AA, Hufford MR (2008). Ecological momentary assessment. Annu Rev Clin Psychol.

[41] Petrie KJ, Jago LA, Devcich DA (2007). The role of illness perceptions in patients with medical conditions. Curr Opin Psychiatry.

[42] Wendrich K, van Oirschot P, Martens MB, Heerings M, Jongen PJ, Krabbenborg L (2019). Toward digital self-monitoring of multiple sclerosis: investigating first experiences, needs, and wishes of people with MS. Int J MS Care.

[43] Pennebaker J, Chung CK (2007). Expressive writing, emotional upheavals, and health. Foundations of Health Psychology.

[44] Greenhalgh J, Ford H, Long AF, Hurst K (2004). The MS Symptom and Impact Diary (MSSID): psychometric evaluation of a new instrument to measure the day to day impact of multiple sclerosis. J Neurol Neurosurg Psychiatry.

[45] Hellard ME, Sinclair MI, Forbes AB, Fairley CK (2001). A randomized, blinded, controlled trial investigating the gastrointestinal health effects of drinking water quality. Environ Health Perspect.

[46] Zhao Y, Ni Q, Zhou R (2018). What factors influence the mobile health service adoption? A meta-analysis and the moderating role of age. Intl J Inform Manag.

[47] Stone AA, Shiffman S, Schwartz JE, Broderick JE, Hufford MR (2003). Patient compliance with paper and electronic diaries. Control Clin Trials.

[48] Zhang A, Xing L, Zou J, Wu JC (2022). Shifting machine learning for healthcare from development to deployment and from models to data. Nat Biomed Eng.

[49] Koops S, Brederoo SG, de Boer JN, Nadema FG, Voppel AE, Sommer IE (2021). Speech as a biomarker for depression. CNS Neurol Disord Drug Targets.

[50] Wolf T, Debut L, Sanh V, Chaumond J, Delangue C, Moi A, Cistac P, Rault T, Louf R, Funtowicz M, Davison J, Shleifer S, von PP, Ma C, Jernite Y, Plu J, Xu C, Le ST, Gugger S, Drame M, Lhoest Q, Rush A (2020). Transformers: state-of-the-art natural language processing. Proceedings of the 2020 Conference on Empirical Methods in Natural Language Processing: System Demonstrations.

[51] Thorpe J, Forchhammer BH, Maier AM (2019). Adapting mobile and wearable technology to provide support and monitoring in rehabilitation for dementia: feasibility case series. JMIR Form Res.

[52] Cornet VP, Holden RJ (2018). Systematic review of smartphone-based passive sensing for health and wellbeing. J Biomed Inform.

