# Supplementary material for: Electronic Health Diary Campaigns to Complement Longitudinal Assessments in Persons With Multiple Sclerosis: Nested Observational Study
Source: JMIR Mhealth Uhealth. 2022 Oct 5;10(10):e38709. doi: 10.2196/38709 (PMC9582921; doi:10.2196/38709)
Supplement: Multimedia Appendix 1 [file mhealth_v10i10e38709_app1.docx]

**Multimedia Appendix 1. Electronic health diary of the Swiss Multiple Sclerosis Registry.**

**
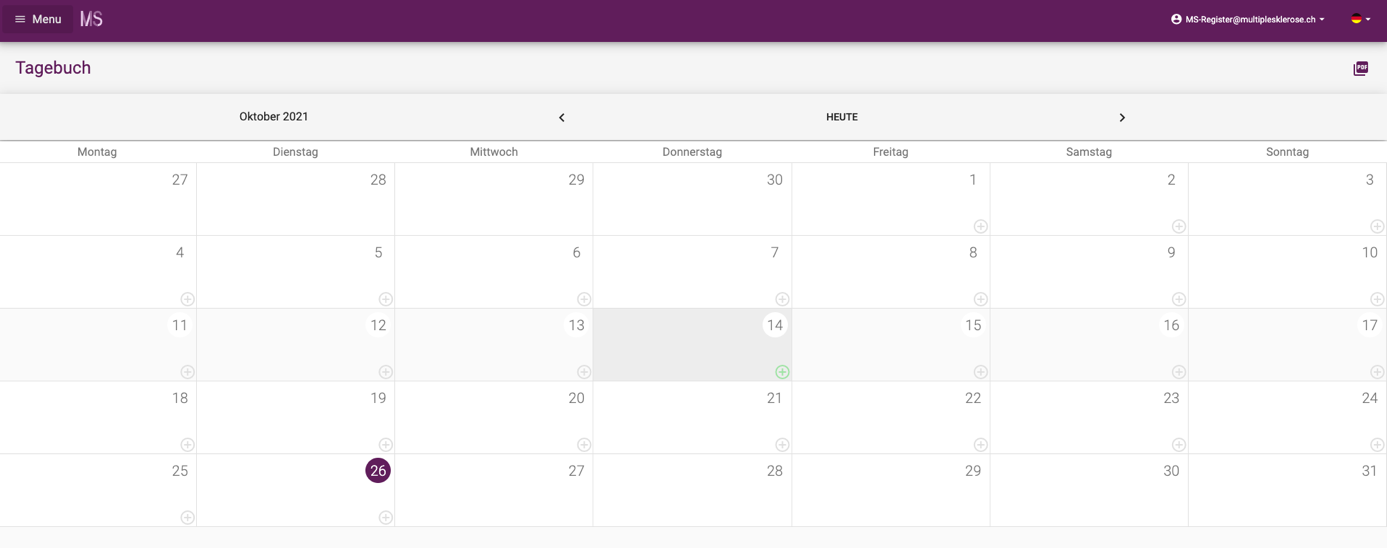
Figure S1.** Home page of the electronic health diary. Monthly overview, where a day can be chosen to create a diary entry.


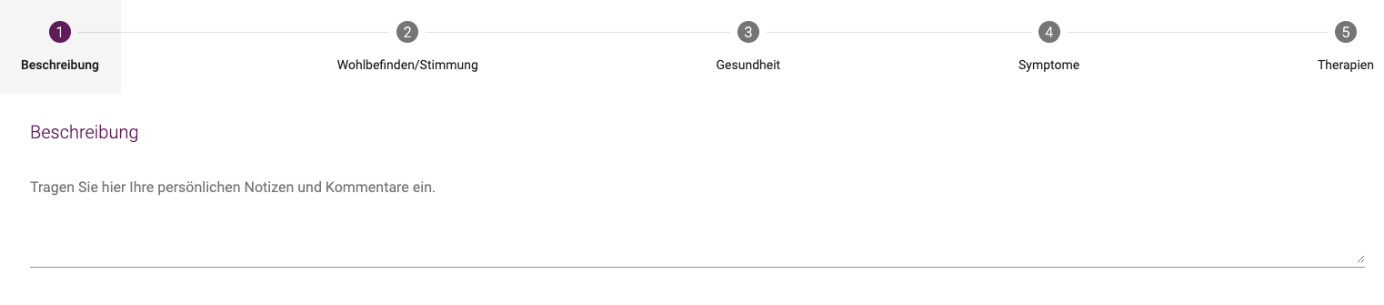
**Figure S2.** First diary question. Possibility to write freely about a topic of choice (e.g., well-being, a particular event of the day).


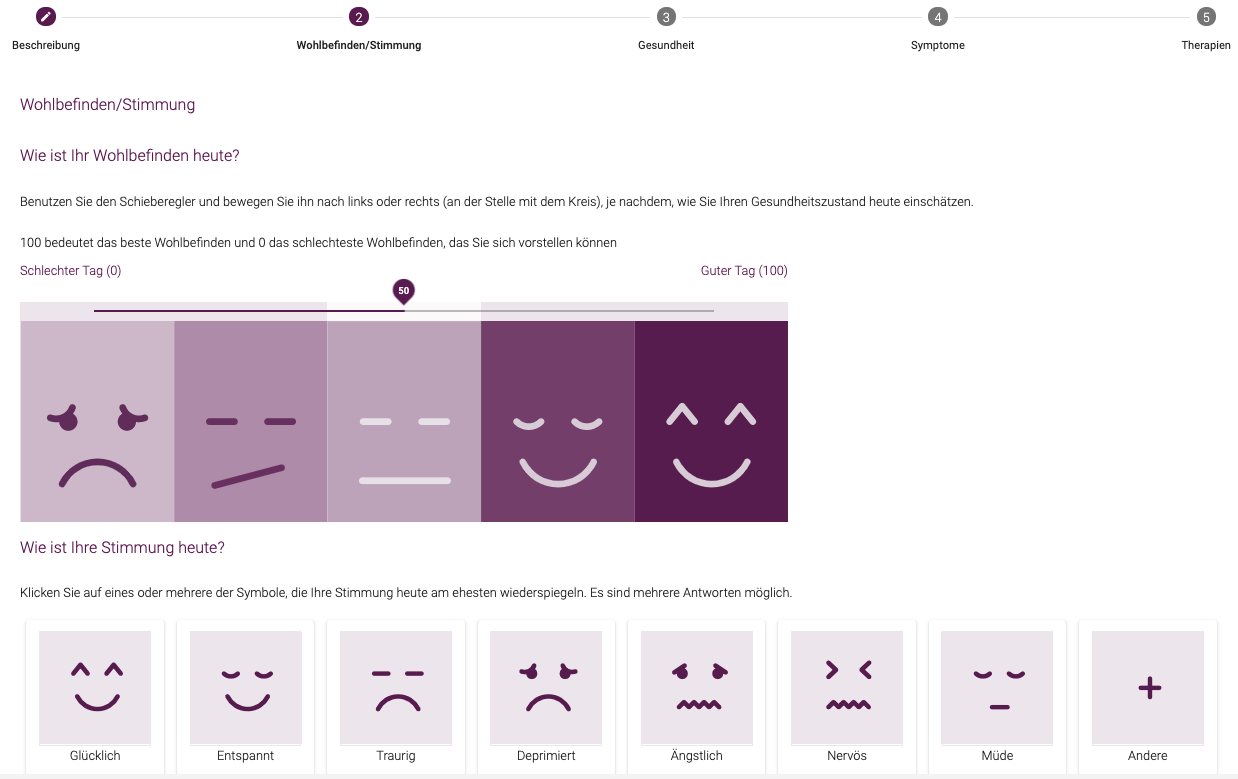
**Figure S3.** Second diary question. The diary’s participants have the possibility to share how they feel (using the EQ-VAS scale) and their mood.

**Figure S4.** Third diary question. EQ-5D-5L questionnaire.


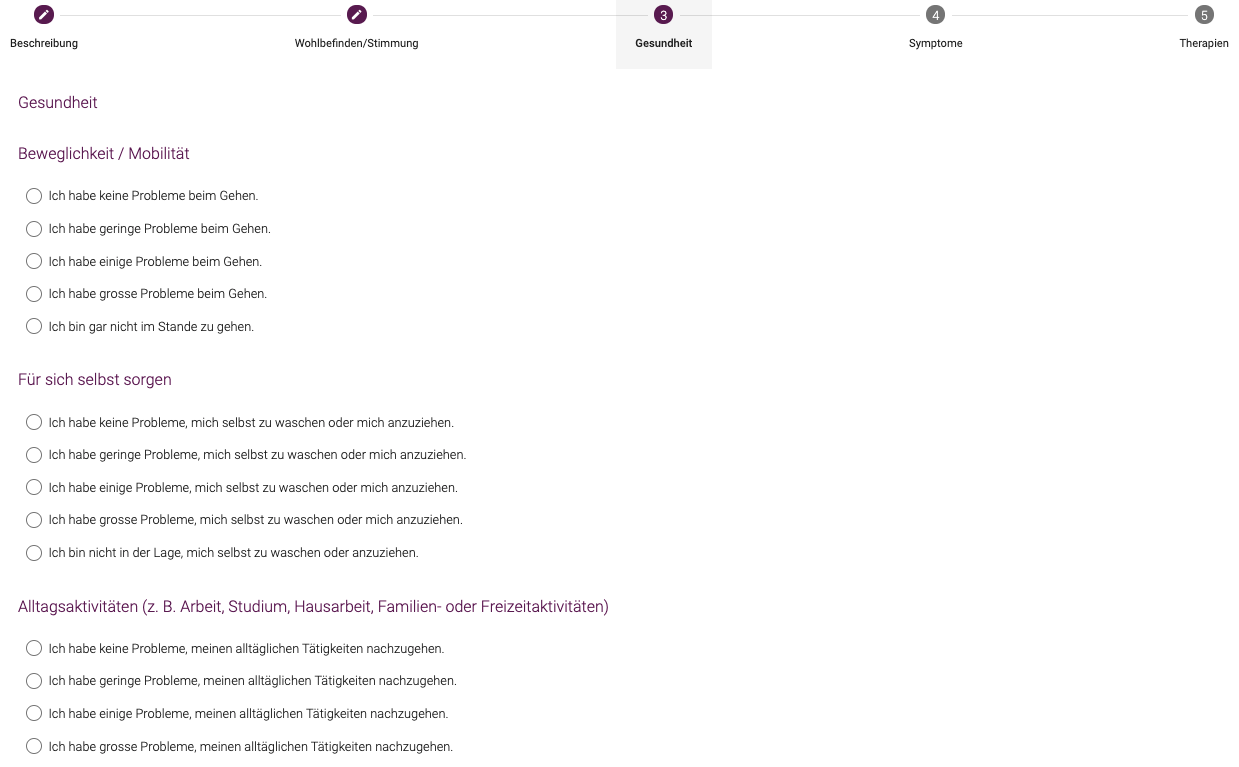

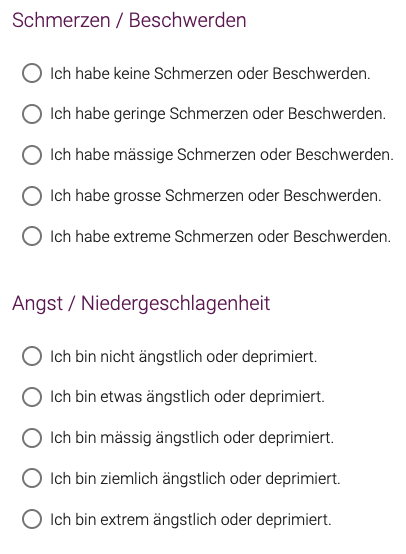


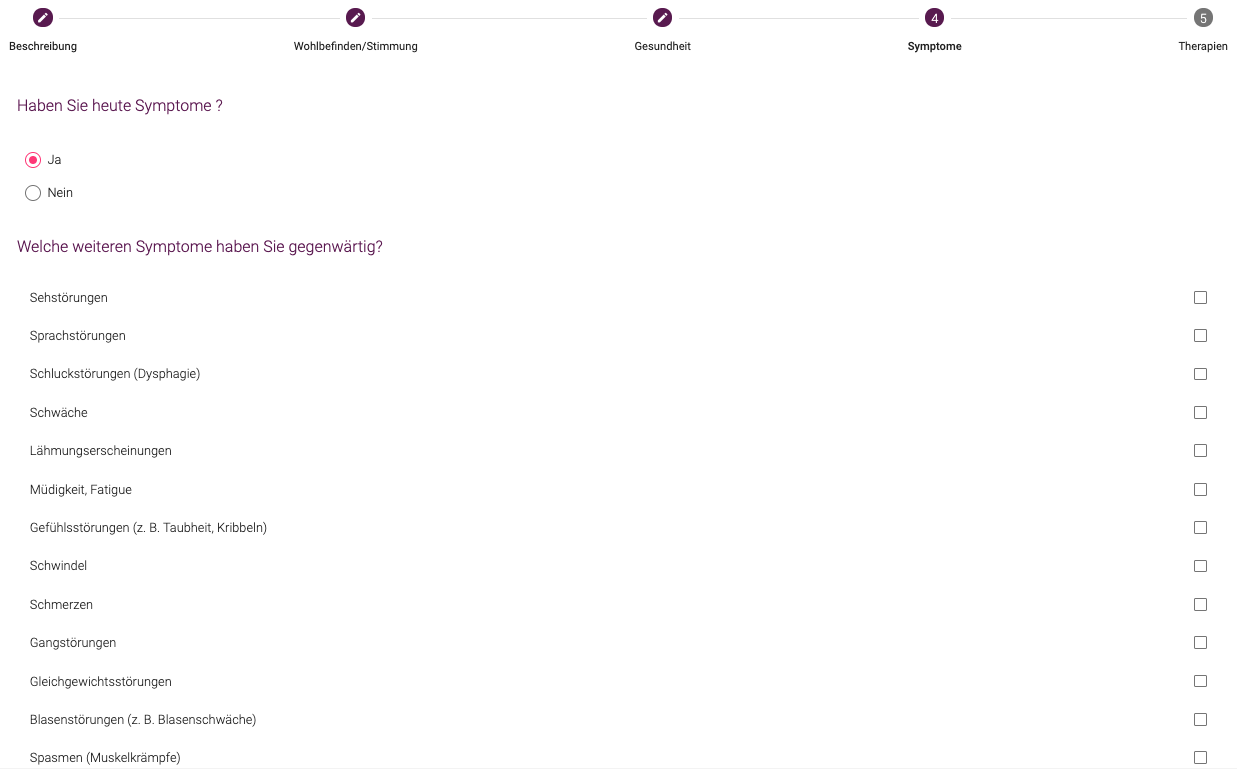
**Figure S5.** Fourth diary question. Diary participants can say if they have symptoms on that day and, if yes, which one (checkboxes).

**Figure S6.** Fifth diary question. Diary participants can select the disease-modifying treatment they are currently taking if any. They can also mention which alternative medicine treatments they use.

Aktuelle Liste Immunmodulierende Medikamente (=spezifische MS-Medikamente)

Betaferon® / Interferon beta 1b

Extavia® / Interferon beta 1b

Avonex® / Interferon beta 1a

Rebif® / Interferon beta 1a

Plegridy® / Peginterferon beta 1a

Copaxone® / Glatirameracetat

Gilenya® / Fingolimod

Tysabri® / Natalizumab

Tecfidera® / BG-12

Aubagio® / Teriflunomid

Nerventra® / Laquinimod

Lemtrada™ / Alemtuzumab

Novantron® / Mitoxantron

Imurek® / Azathioprin

Synacthen® / Corticotropin

Sandimmun® / Cyclosporin

Endoxan® / Cyclophosphamid

MabThera® / Rituximab

Ocrevus® / Ocrelizumab

Anderes Medikament 1 ________________________

Anderes Medikament 2 ________________________

Alternativmedizinische Behandlungen oder Medikamente

(Keine)

Naturheilkunde

Osteopathie

TCM (Traditionelle Chinesische Medizin)

Entspannungstherapien (Progressive Muskelrelaxation, Yoga, Qigong, Tai-Chi)

Homöopathie

Akupunktur

Triggerpunkttherapie

Aromatherapie

Vitamin-B-Präparate

Weitere Vitamin- und Mineralstoff-Präparate (z. B. Vitamin C, D, E, Magnesium)

Preiselbeersaft

Andere

Falls andere Behandlungen oder Medikamente, welche?

_____________________________________________

_____________________________________________

_____________________________________________
