# Supplementary material for: Electronic Health Diary Campaigns to Complement Longitudinal Assessments in Persons With Multiple Sclerosis: Nested Observational Study
Source: JMIR Mhealth Uhealth. 2022 Oct 5;10(10):e38709. doi: 10.2196/38709 (PMC9582921; doi:10.2196/38709)
Supplement: Multimedia Appendix 2 [file mhealth_v10i10e38709_app2.docx]

**Multimedia Appendix 2. Invitation letters’ to the electronic health diary campaign for the Swiss Multiple Sclerosis Registry participants.**

**Teaser Newsletter (end of January 2019)**

A week in the life of...

The first diary campaign of the Swiss MS Registry is about ‘**A week in the life of people with MS**’, which will take place from **09 to 17 March 2019**.

At the Swiss MS Registry, the topic of quality of life in everyday life is at the forefront of research projects. How do people with MS cope with everyday life? What about their physical and psychological well-being? And what factors may promote or hinder quality of life? During this diary week, the Swiss MS Registry would like to invite as many people with MS as possible to explore these questions together and learn from each other thanks to the large knowledge community.

Thereby two goals are in the foreground:

On the one hand, a realistic picture of the quality of life of people with MS should be created, which can also be brought to the public. On the other hand, the knowledge of those people with MS as MS experts for their own well-being is to be documented and made accessible in anonymous form to other people affected by MS.

The data collected during this week will be processed and evaluated by the MS Registry Data Center at the University of Zurich and made available online as a final report.

More information about the diary action "A week in the life of..." will be available soon.

**Guidance by email to MSR participants (28.02.2019)**

Under the motto "A week in the life of..." the Swiss MS Registry launches the first diary action.

What is it about?

The first diary action is about the theme ‘**A week in the life of people with MS**’. The Swiss MS Registry and the Swiss MS Society would like to hear from as many people with MS as possible during one week about how they feel physically and psychologically and what measures people with MS can take to positively influence their well-being. With your diary entries on the MS Register platform, you contribute together with other people affected to a lively exchange of experiences in our research community.

How does it work?

Log in to the MS Registry platform, click on "My Diary" in the menu and start your entry. Using your diary entries, you will help us understand questions about well-being, symptoms, and medical and complementary treatments. Tell us what has positively or negatively affected your well-being and quality of life during your week and be part of creating a comprehensive picture of the everyday life of people with MS for the first time from March 09 to 17, 2019.

What happens to my data?

The data collected during this week will be promptly processed by the MS Registry Data Center at the University of Zurich, evaluated, and made available in anonymized form as a final report. In order to promote the exchange of experiences with other affected persons, we would like to use selected entries from the comment field as quotations in the final report. Of course, the MS Registry adheres to all legal rules of data protection. The data is collected and stored in a secure, password-protected environment. When evaluating the data and reproducing quotes, it is ensured that absolutely no conclusions can be drawn about your person.

With the data thus obtained, the MS Registry hopes to gain even more insight into which factors can positively or negatively influence the daily lives of people with MS. These findings are of great importance for people with MS and health professionals a great deal of quality of life can be regained with targeted measures

By the way:

Since last December, the diary on the MS Register platform can also be downloaded and printed out as a PDF and thus serve, for example, as a possible reminder for your next visit to the doctor. In addition, the functions have been significantly revised with the help of the MS Register Board and thus better adapted to the needs of those affected. Input aids for complementary medicine and symptomatic treatments have been added and thanks to a calendar function, past events can also be documented in the diary. All entries can be viewed on an overview page. The diary can therefore also be a helpful support for you after the diary action week.

We look forward to your participation!
