# Supplementary figures and images for: Electronic Health Diary Campaigns to Complement Longitudinal Assessments in Persons With Multiple Sclerosis: Nested Observational Study
Source: JMIR Mhealth Uhealth. 2022 Oct 5;10(10):e38709. doi: 10.2196/38709 (PMC9582921; doi:10.2196/38709)

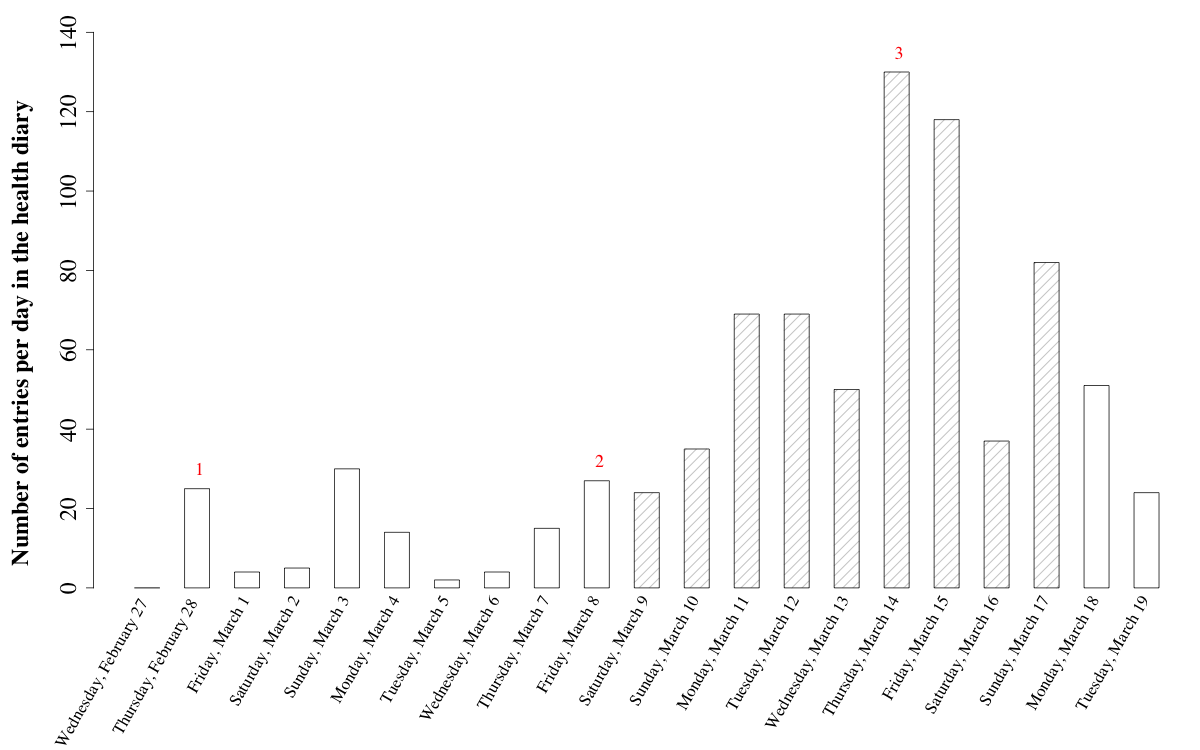

Supplement: Multimedia Appendix 3 [file mhealth_v10i10e38709_app3.png]

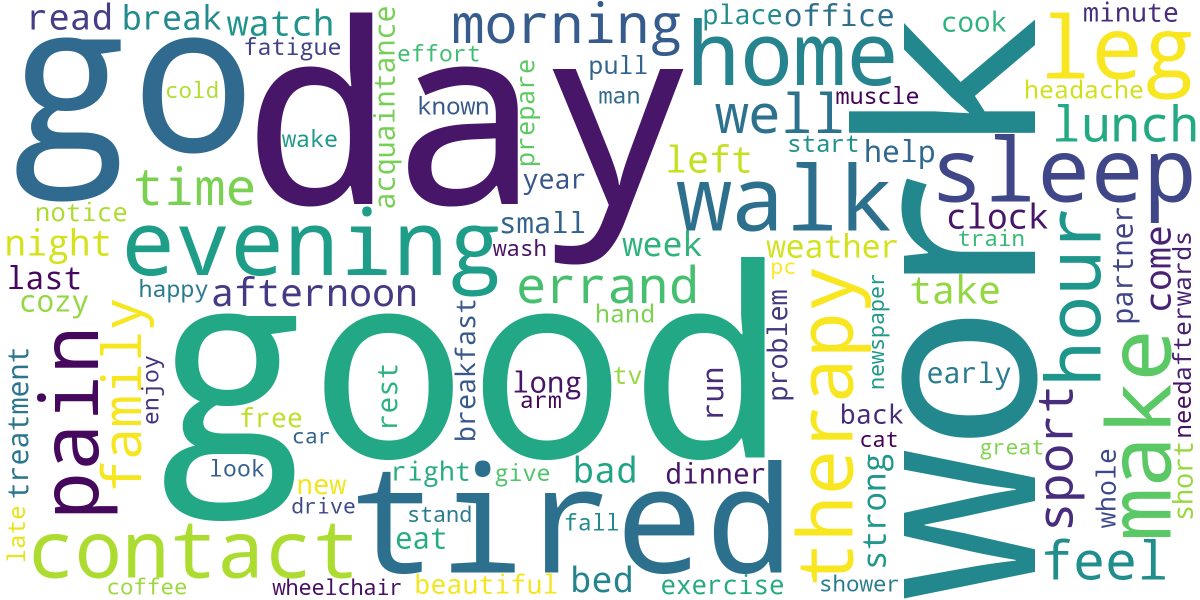

Supplement: Multimedia Appendix 7 [file mhealth_v10i10e38709_app7.png]
