# Supplementary material for: Electronic Health Diary Campaigns to Complement Longitudinal Assessments in Persons With Multiple Sclerosis: Nested Observational Study
Source: JMIR Mhealth Uhealth. 2022 Oct 5;10(10):e38709. doi: 10.2196/38709 (PMC9582921; doi:10.2196/38709)

**Multimedia Appendix 4. Flow chart of the Swiss Multiple Sclerosis Registry enrollees and the electronic health diary participants.**

**Figure S1.** Overall study flow displaying, on the left side of the red broken line, the selection of the study population based on the electronic health diary entries they wrote during the study period (February 27, 2019 to March 19, 2019), and on the right side of the red broken line, the study participants selection based on the completion of the baseline and follow-up surveys offered by the Swiss Multiple Sclerosis Registry (already represented in Figure 1).

**Left side of the red broken line**: Two hundred fifty-one persons with multiple sclerosis used the electronic health diary between February 27, 2019, and March 19, 2019, creating a total of 1411 diary entries. Duplicates diary entries (n=9) were deleted as well as the entries in which the free text field section was left blank (n=317). By removing participants who had not completed the baseline assessments by March 26, 2019, and the follow-up survey (shown on the right side of the red broken line), we lost 270 entries. In total, 815 health diary entries created by 134 participants were available for analysis. However, to conduct the LIWC analysis, entries not written in German were discarded (n=183), leaving 632 entries written by 100 participants for the analysis. For the word cloud creation, entries of less than 10 words were removed (n=106). Finally, 526 entries written by 93 participants were available for the word cloud.

**Right side of the red broken line**: In the Swiss Multiple Sclerosis Registry, 1550 persons with multiple sclerosis completed baseline assessments. Of those, 1318 participated through the Swiss Multiple Sclerosis Registry web platform. After the application of inclusion criteria and data quality checks, 1274 online enrollees with baseline assessments completed before March 26, 2019 were included. Of those, 658 enrollees had completed the follow-up survey, and among them, 134 had made a non-empty free text entry into the electronic health diary within the analysis time frame (February 27, 2019, and March 19, 2019).

The **bold one-sided arrow** connecting both sides of the flow chart represents the link existing between the 2 population samples compared in this study. Out of the 658 enrollees in the Swiss Multiple Sclerosis Registry with completed the baseline assessments, 134 (20.4 %) used the health diary between February 27, 2019, and March 19, 2019, resulting in 815 diary entries.

N_P1_ or n_P1_: number of Swiss Multiple Sclerosis Registry enrollees with completed baseline assessments

N_P2_ or n_P2_: number of Swiss Multiple Sclerosis Registry enrollees with health diary campaign data

N_D_ or n_D_: number of health diary campaign entries


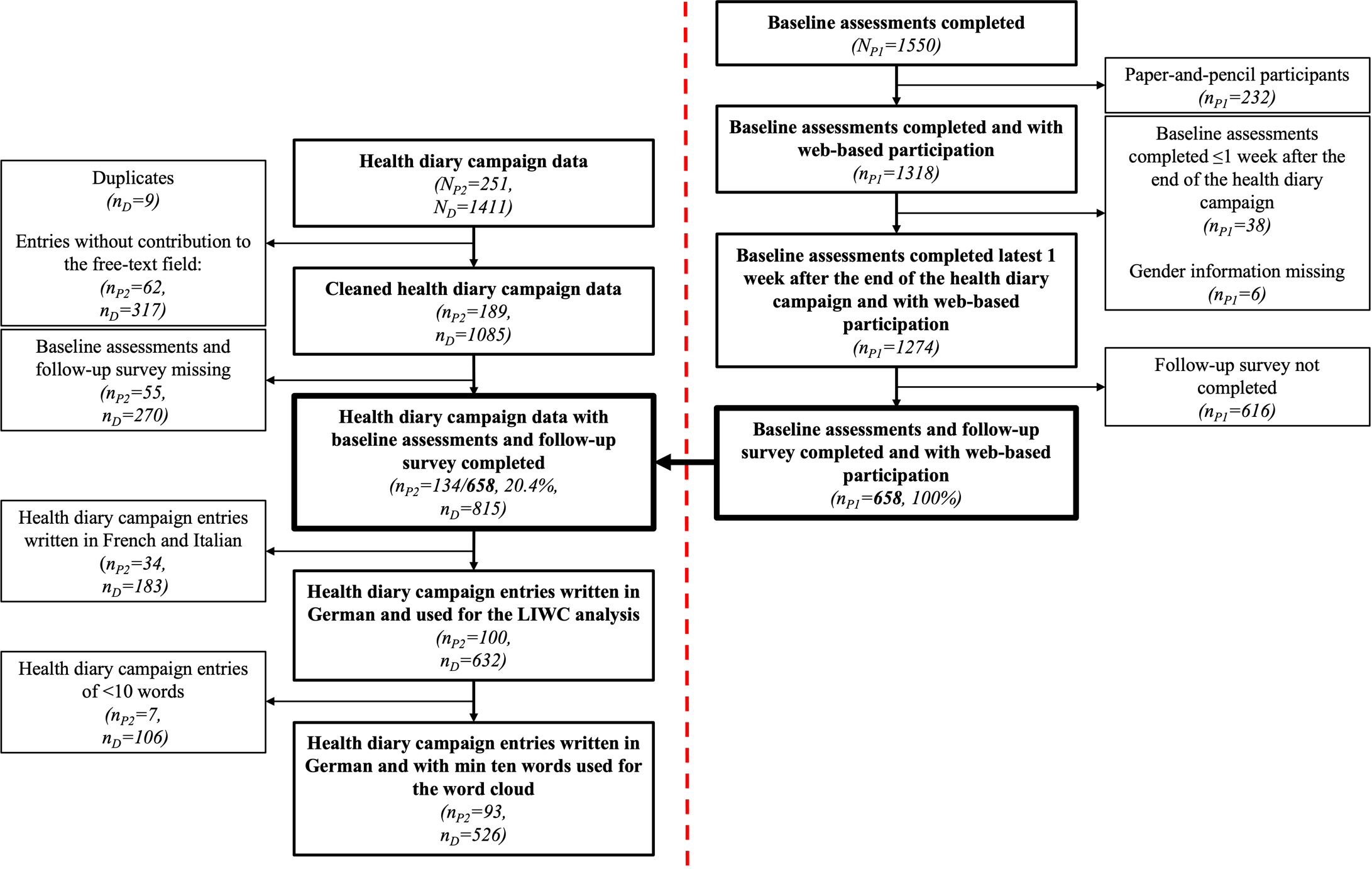

Supplement: Multimedia Appendix 4 [file mhealth_v10i10e38709_app4.docx]
