# Supplementary material for: Electronic Health Diary Campaigns to Complement Longitudinal Assessments in Persons With Multiple Sclerosis: Nested Observational Study
Source: JMIR Mhealth Uhealth. 2022 Oct 5;10(10):e38709. doi: 10.2196/38709 (PMC9582921; doi:10.2196/38709)
Supplement: Multimedia Appendix 5 [file mhealth_v10i10e38709_app5.docx]

**Multimedia Appendix 5. Full list of the symptoms experienced within the last 12 months and the disease-modifying medications used within the last 6 months.**

**Table S1.** Complementary information to Table 1: More detailed information about the study population (N = 658) regarding their symptoms within the last 12 months and their disease-modifying medications within the last 6 months and their *P* value (significance level: 0.05).^a^

| **Characteristics** | **Participants in health diary campaign**  **(n = 134)** | **Non-participants**  **(n = 524)** | ***P* value**  **(Chi-square test)** |
| --- | --- | --- | --- |
| **Symptoms experienced within the last twelve months^b^, n (%)** |  |  | .25 |
| None | 50 (37.3%) | 227 (43.3%) |  |
| Fatigue | 57 (42.5%) | 210 (40.1%) |  |
| Gait disorder | 52 (38.8%) | 138 (26.3%) |  |
| Paresthesia (eg, numbness, tingling) | 50 (37.3%) | 187 (35.7%) |  |
| Spasms (muscle cramps) | 45 (33.6%) | 119 (22.7%) |  |
| Vestibular disorders | 43 (32.1%) | 135 (25.8%) |  |
| Weakness | 40 (29.9%) | 129 (24.6%) |  |
| Pain | 40 (29.9%) | 143 (27.3%) |  |
| Bladder disorders (eg, bladder weakness) | 40 (29.9%) | 109 (20.8%) |  |
| Concentration problems | 35 (26.1%) | 127 (24.2%) |  |
| Intestinal disorders (eg, constipation) | 29 (21.6%) | 83 (15.8%) |  |
| Dizziness | 20 (14.9%) | 74 (14.1%) |  |
| Visual impairments | 19 (14.2%) | 65 (12.4%) |  |
| Signs of paralysis | 19 (14.2%) | 51 (9.7%) |  |
| Memory disorders | 19 (14.2%) | 85 (16.2%) |  |
| Tremor | 18 (13.4%) | 41 (7.8%) |  |
| Sexual disorders | 17 (12.7%) | 63 (12%) |  |
| Speech disorders | 16 (11.9%) | 25 (4.8%) |  |
| Convulsions, tics | 16 (11.9%) | 42 (8%) |  |
| Swallowing difficulties (dysphagia) | 11 (8.2%) | 26 (5%) |  |
| Depression | 10 (7.5%) | 49 (9.4%) |  |
| Affective lability, lack of control over emotions | 10 (7.5%) | 30 (5.7%) |  |
| Problems with spatial orientation | 9 (6.7%) | 16 (3.1%) |  |
| Other | 3 (2.2%) | 3 (0.6%) |  |
| Epileptic convulsions | 1 (0.7%) | 2 (0.4%) |  |
| **Disease-modifying medications used within the last six months^b^, n (%)** |  |  | NA^c^ |
| None | 53 (39.6%) | 156 (29.8%) |  |
| Ocrelizumab (Ocrevus) | 28 (20.9%) | 87 (16.6%) |  |
| Fingolimod (Gilenya) | 15 (11.2%) | 90 (17.2%) |  |
| Dimethyl fumarate (Tecfidera) | 10 (7.5%) | 54 (10.3%) |  |
| Interferon beta-1a (Rebif) | 6 (4.5%) | 19 (3.6%) |  |
| Glatiramer acetate (Copaxone) | 6 (4.5%) | 18 (3.4%) |  |
| Interferon beta-1b (Betaferon) | 5 (3.7%) | 14 (2.7%) |  |
| Teriflunomide (Aubagio) | 5 (3.7%) | 15 (2.9%) |  |
| Natalizumab (Tysabri) | 3 (2.2%) | 40 (7.6%) |  |
| Rituximab (MabThera) | 3 (2.2%) | 15 (2.9%) |  |
| Interferon beta-1a (Avonex) | 2 (1.5%) | 10 (1.9%) |  |
| Peginterferon beta-1a (Plegridy) | 1 (0.7%) | 4 (0.8%) |  |
| Mitoxantrone (Novantron) | 1 (0.7%) | 0 |  |
| Other | 1 (0.7%) | 8 (1.5%) |  |
| Interferon beta-1b (Extavia) | 0 | 0 |  |
| Laquinimod (Nerventra) | 0 | 0 |  |
| Alemtuzumab (Lemtrada) | 0 | 0 |  |
| Azathioprine (Imurek) | 0 | 0 |  |
| Tetracosactide (Synacthen) | 0 | 0 |  |
| Cyclosporine (Sandimmun) | 0 | 0 |  |
| Cyclophosphamide (Endoxan) | 0 | 0 |  |
| Cladribin (Mavenclad) | 0 | 2 (0.4%) |  |
| Siponimod (Mayzent) | 0 | 0 |  |
| Ofatumumab (Arzerra) | 0 | 0 |  |
| ^a^The table below reports all symptoms and disease-modifying medication options study participants could indicate in the baseline assessments.  ^b^Multiple answers possible.  ^c^NA: The Chi-square test and its *P* value could not be computed due to expected frequencies being zeros. | | | |
