# Supplementary material for: Electronic Health Diary Campaigns to Complement Longitudinal Assessments in Persons With Multiple Sclerosis: Nested Observational Study
Source: JMIR Mhealth Uhealth. 2022 Oct 5;10(10):e38709. doi: 10.2196/38709 (PMC9582921; doi:10.2196/38709)
Supplement: Multimedia Appendix 6 [file mhealth_v10i10e38709_app6.docx]

**Multimedia Appendix 6. Completeness of the diary entries.**

**Table S1.** Frequency counts and percentages of diary entries and completeness of specific diary sections for 134 participants in the electronic health diary campaign running between February 27, 2019, and March 19, 2019.^a^

| **Categories** | **Total number of possible entries** | **Number of entries written, n (%)** |
| --- | --- | --- |
| **Free text field** completed | 815 | 815 (100%) |
| **Well-being** description completed | 815 | 528 (64.8%) |
| **Mood** description completed | 815 | 681 (83.6%) |
| **EQ-5D**^b^ section completed | 815 | 633 (77.7%) |
| **Symptoms** section completed | 815 | 644 (79%) |
| Have symptoms  (among “Symptoms section completed”) | 644 | 400 (62.1%) |
| At least one symptom given  (among “Have symptoms”) | 400 | 398 (99.5%) |
| **Treatment** section completed | 815 | 616 (75.6%) |
| Follow a treatment  (among “Treatment section completed”) | 616 | 541 (87.8%) |
| At least one treatment given  (among “Follow a treatment”) | 541 | 534 (98.7%) |
| Treatment adherence section completed | 815 | 572 (70.2%) |
| Treatment forgotten  (among “Treatment adherence section completed”) | 572 | 14 (2.4%) |
| ^a^Total number of entries written during this period: N=815. The order of categories is the same as presented in the electronic health diary.  ^b^EQ-5D: EuroQol 5-Dimension. | | |
