# Supplementary material for: Electronic Health Diary Campaigns to Complement Longitudinal Assessments in Persons With Multiple Sclerosis: Nested Observational Study
Source: JMIR Mhealth Uhealth. 2022 Oct 5;10(10):e38709. doi: 10.2196/38709 (PMC9582921; doi:10.2196/38709)
Supplement: Multimedia Appendix 8 [file mhealth_v10i10e38709_app8.docx]

**Multimedia Appendix 8. Word frequency of the 25 most frequent words of the word cloud.**


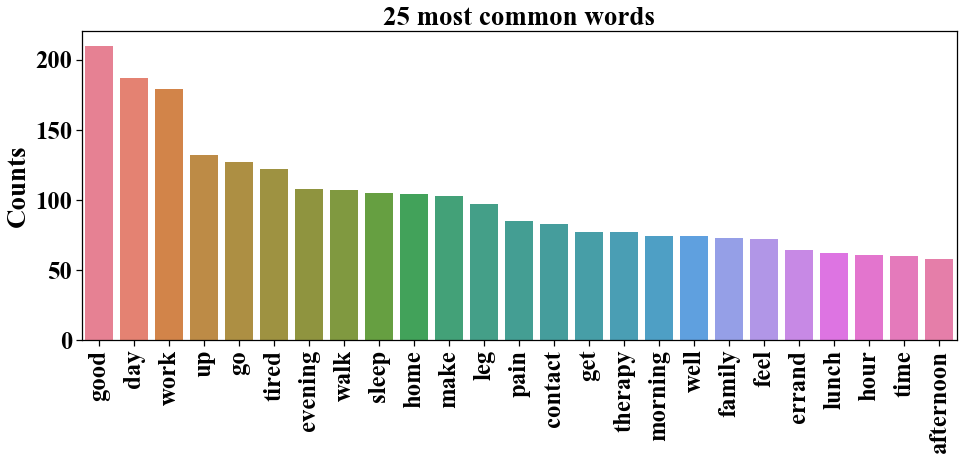
**Figure S1.** Bar chart displaying the 25 most used words throughout the 526 electronic health diary entries and their frequency. This graph is a quantitative replication of the word cloud presented in Figure 4.
