# Supplementary material for: Electronic Health Diary Campaigns to Complement Longitudinal Assessments in Persons With Multiple Sclerosis: Nested Observational Study
Source: JMIR Mhealth Uhealth. 2022 Oct 5;10(10):e38709. doi: 10.2196/38709 (PMC9582921; doi:10.2196/38709)
Supplement: Multimedia Appendix 9 [file mhealth_v10i10e38709_app9.docx]

**Multimedia Appendix 9. Word cloud of 100 words and word frequency of the 25 most frequent words throughout the electronic health diary entries per multiple sclerosis type.**


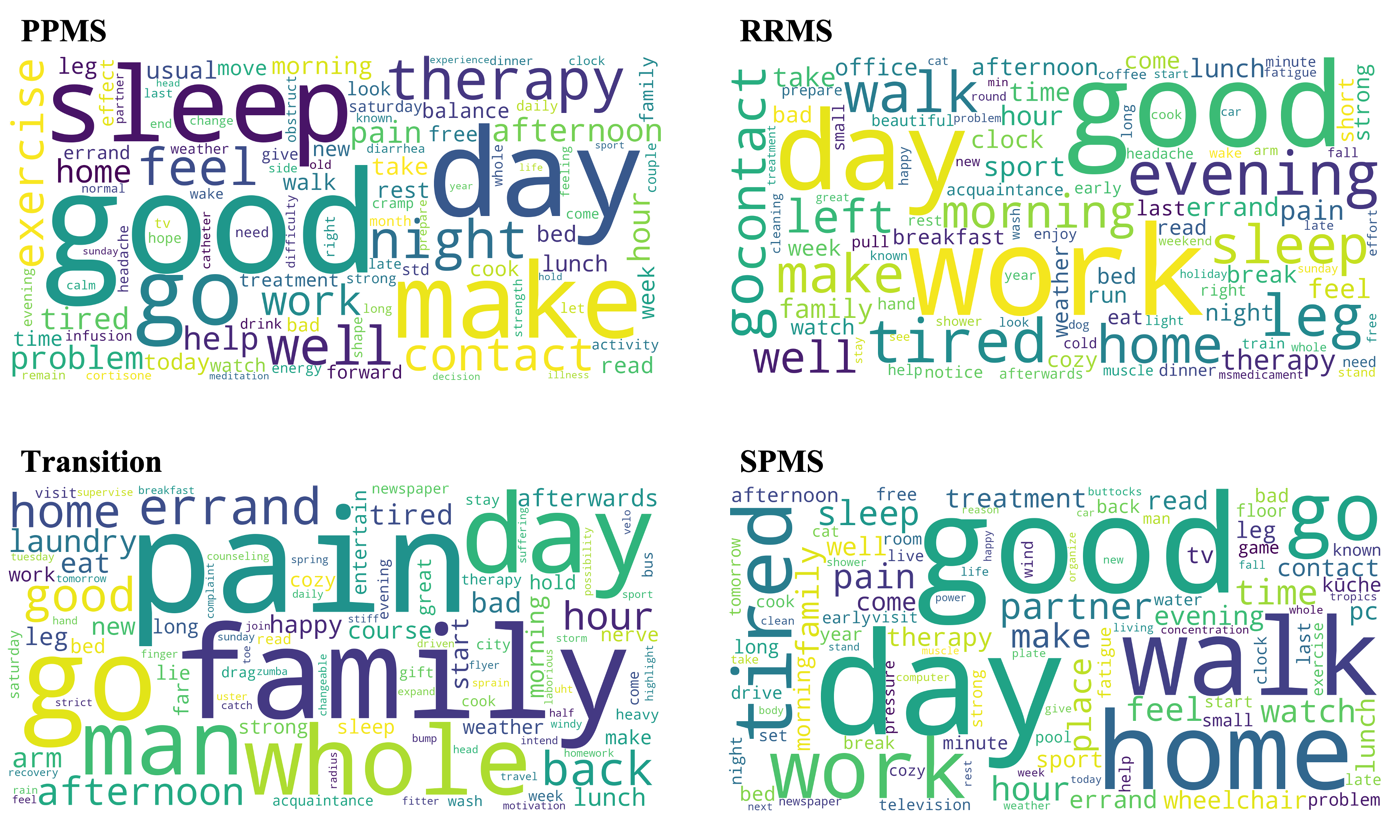
**Figure S1.** Word cloud per MS type (ie, primary progressive multiple sclerosis or PPMS, relapsing-remitting multiple sclerosis or RRMS, transition, and secondary progressive multiple sclerosis or SPMS) of the 100 most frequent words appearing in electronic health diary entries of a minimum length of 10 words (n=61, n=348, n=11, and n=106, respectively). The font size reflects the frequency of words’ occurrence.


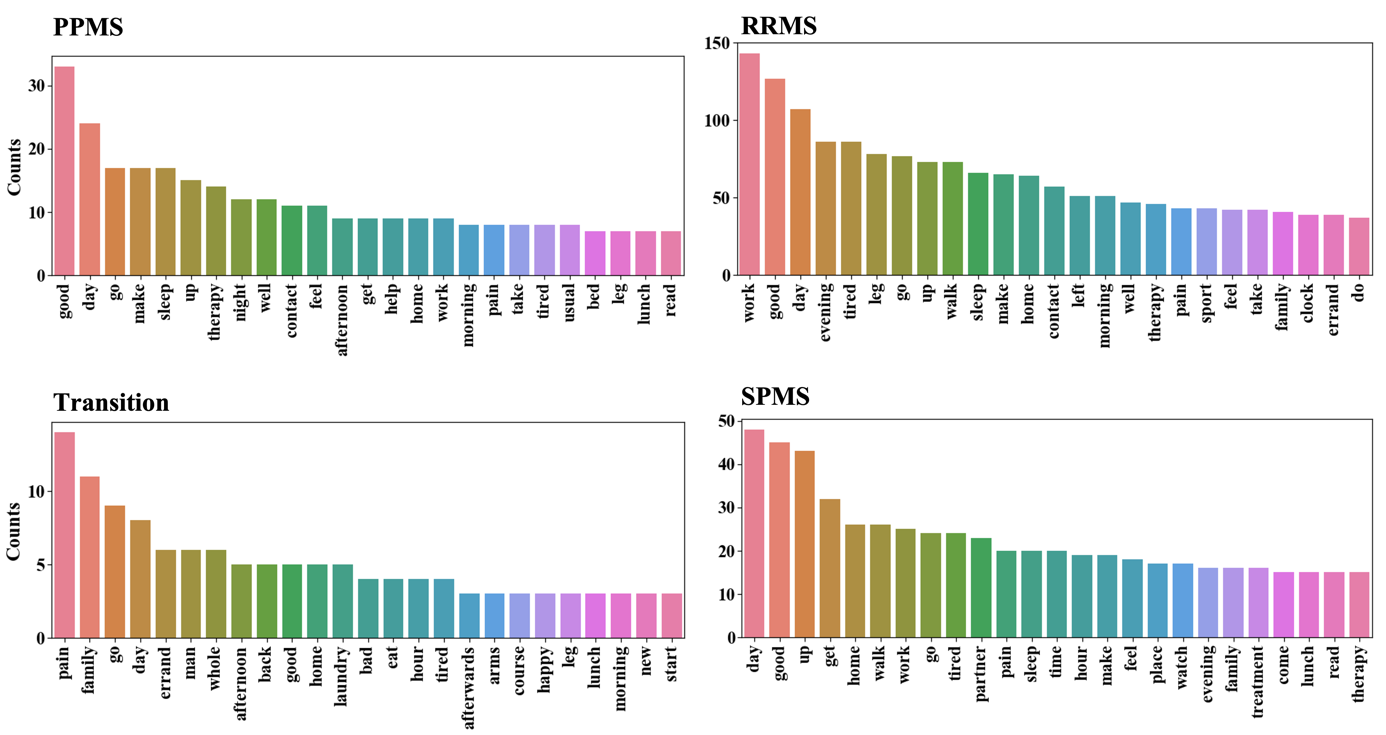
**Figure S2.** Bar chart per MS type (ie, primary progressive multiple sclerosis or PPMS, relapsing-remitting multiple sclerosis or RRMS, transition, and secondary progressive multiple sclerosis or SPMS) displaying the 25 most used words throughout the electronic health diary entries (n=61, n=348, n=11, and n=106, respectively) and their frequency. This graph is a quantitative replication of the word clouds presented in Figure S1, Multimedia Appendix 7.
