# Supplementary material for: Electronic Health Diary Campaigns to Complement Longitudinal Assessments in Persons With Multiple Sclerosis: Nested Observational Study
Source: JMIR Mhealth Uhealth. 2022 Oct 5;10(10):e38709. doi: 10.2196/38709 (PMC9582921; doi:10.2196/38709)
Supplement: Multimedia Appendix 10 [file mhealth_v10i10e38709_app10.docx]

**Multimedia Appendix 10. Jaccard indices of the full disease-modifying medication list.**

**Table S1.** Similarity between diary and follow-up survey information on medication use provided by 134 electronic health diary campaign participants.^a^

| **Characteristics** | **Overlap** | **Union** | **Jaccard Index** |
| --- | --- | --- | --- |
| **Disease-modifying medications** |  |  |  |
| Interferon beta-1a (Avonex) | 2 | 2 | 1 |
| Glatiramer acetate (Copaxone) | 5 | 7 | 0.71 |
| Fingolimod (Gilenya) | 12 | 21 | 0.57 |
| Teriflunomide (Aubagio) | 4 | 8 | 0.5 |
| Interferon beta-1b (Betaferon) | 2 | 5 | 0.4 |
| Dimethyl fumarate (Tecfidera) | 1 | 32 | 0.03 |
| Ocrelizumab (Ocrevus) | 0 | 36 | 0 |
| Other | 0 | 21 | 0 |
| Cyclosporine (Sandimmun) | 0 | 10 | 0 |
| Interferon beta-1a (Rebif) | 0 | 6 | 0 |
| Natalizumab (Tysabri) | 0 | 6 | 0 |
| Rituximab (MabThera) | 0 | 3 | 0 |
| Peginterferon beta-1a (Plegridy) | 0 | 1 | 0 |
| Alemtuzumab (Lemtrada) | 0 | 1 | 0 |
| Mitoxantrone (Novantron) | 0 | 1 | 0 |
| Tetracosactide (Synacthen) | 0 | 1 | 0 |
| Interferon beta-1b (Extavia) | 0 | 0 | 0 |
| Laquinimod (Nerventra) | 0 | 0 | 0 |
| Azathioprine (Imurek) | 0 | 0 | 0 |
| Cyclophosphamide (Endoxan) | 0 | 0 | 0 |
| ^a^The Jaccard index was used to measure the similarity (reporting overlap) among the different sources (0=no similarity, 1=maximum similarity). The column *Overlap* represents the number of participants with overlapping reports in the diary and in the follow-up survey. The column *Union* represents the total number of participants who reported a specific data item at least once in the diary or the follow-up survey collecting the symptoms experienced within the last 12 months and the medication used within the last 6 months. The column *Jaccard* *Index* represents the quotient of the *Overlap* values and the *Union* values. | | | |
